# Oral health and cardiovascular care: Perceptions of people with cardiovascular disease

**DOI:** 10.1371/journal.pone.0181189

**Published:** 2017-07-20

**Authors:** Paula Sanchez, Bronwyn Everett, Yenna Salamonson, Shilpi Ajwani, Sameer Bhole, Joshua Bishop, Karen Lintern, Samantha Nolan, Rohan Rajaratnam, Julie Redfern, Maria Sheehan, Fiona Skarligos, Lissa Spencer, Ravi Srinivas, Ajesh George

**Affiliations:** 1 School of Nursing and Midwifery, Western Sydney University, Sydney, Australia; 2 South Western Sydney Local Health District, Sydney, Australia; 3 Collaboration for Oral Health Outcomes, Research Translation and Evaluation (COHORTE) Research Group, Liverpool, Australia; 4 Ingham Institute for Applied Medical Research, Liverpool, Australia; 5 Centre for Applied Nursing Research, Liverpool, Australia; 6 Sydney Local Health District, Sydney, Australia; 7 Oral Health Services, Sydney Dental Hospital, Sydney, Australia; 8 Faculty of Dentistry, The University of Sydney, Sydney, Australia; 9 School of Medicine, Western Sydney University, Sydney, Australia; 10 University of New South Wales, Sydney, Australia; 11 The George Institute for Global Health, Sydney, Australia; 12 Sydney Medical School, The University of Sydney, Sydney, Australia; University of Tampere, FINLAND

## Abstract

**Main objective:**

The aim of this study was to explore the perception of patients with cardiovascular disease towards oral health and the potential for cardiac care clinicians to promote oral health.

**Method:**

A needs assessment was undertaken with twelve patients with cardiovascular disease attending cardiac rehabilitation between 2015 and 2016, in three metropolitan hospitals in Sydney, Australia. These patients participated in face-to-face semi-structured interviews. Data was analysed using thematic analysis.

**Results:**

Results suggested that while oral health was considered relevant there was high prevalence of poor oral health among participants, especially those from socioeconomic disadvantaged background. Awareness regarding the importance of oral health care its impact on cardiovascular outcomes was poor among participants. Oral health issues were rarely discussed in the cardiac setting. Main barriers deterring participants from seeking oral health care included lack of awareness, high cost of dental care and difficulties in accessing the public dental service. Findings also revealed that participants were interested in receiving further information about oral health and suggested various mediums for information delivery. The concept of cardiac care clinicians, especially nurses providing education, assessment and referrals to ongoing dental care was well received by participants who felt the post-acute period was the most appropriate time to receive oral health care advice. The issues of oral health training for non-dental clinicians and how to address existing barriers were highlighted by participants.

**Relevance to clinical practice:**

The lack of oral health education being provided to patients with cardiovascular disease offers an opportunity to improve care and potentially, outcomes. In view of the evidence linking poor oral health with cardiovascular disease, cardiac care clinicians, especially nurses, should be appropriately trained to promote oral health in their practice. Affordable and accessible dental care services for people with cardiovascular disease should be considered and offered by health services in Australia.

## Introduction

Cardiovascular disease (CVD) is one of the leading causes of chronic disease morbidity and mortality in industrialised countries [1]. According to the World Health Organisation, 31% of all deaths globally are due to CVD [2]. Contributing risk factors of CVD include family history, diabetes, hypertension, hyperlipidaemia, tobacco use, limited physical activity, obesity and poor dietary intake [3]. Additionally, there is also growing evidence that another potential risk factor for CVD is periodontal disease [1, 4, 5].

Periodontal disease is a common chronic inflammatory condition characterised by destruction of the tooth supporting structures due to bacterial infection [6]. The prevalence of periodontal disease is high in the adult population of developed countries [7, 8]. In the USA, the Centre for Disease Control and Prevention reported that between 2009 and 2010, one in every two adults over 30 years had periodontal disease [9]. In Australia, the prevalence rates of periodontitis ranges from 35–53% for those aged above 45 years [10]. Periodontal disease is considered a multifactorial condition where the presence of anaerobic gram-negative bacteria, the person’s immune mechanisms and genetic predisposition are believed to a play an important role [7]. Contributing risk factors include a rapidly aging population, as well as lifestyle factors, such as tobacco use, poor nutrition and stress [9, 11]. Another concerning factor is that in many developing and some developed countries where periodontal health is unsatisfactory, the focus by dental professionals is mainly on treating the condition rather than on prevention periodontal disease [12].

Over the last decade there is increasing evidence linking periodontal disease with CVD and its adverse outcomes [4, 8, 13, 14]. Several studies have demonstrated that inflammatory process reaction in atherosclerotic lesions may be the link between periodontal disease and CVD [5, 15–19]. The current view is that periodontitis leads to entry of bacteria into the circulatory system which in turn activates a host inflammatory response resulting in exacerbation, maturation and, ultimately, atheroma formation thereby increasing the risk of CVD [14, 20]. One study found that among those with established coronary heart disease, periodontal disease increased the likelihood of a recurrent coronary event by nearly 1.5 times [21]. A joint consensus report of the European Federation of Periodontology and American Academy of Periodontology concluded that the evidence supporting an increased risk for future CVD among those with periodontal disease was strong and that the association was independent of other cardiovascular risk factors [14].

A recent Australian study involving 172,630 individuals with CVD concluded that tooth loss and self-rated gum problems were markers for increased risk of ischaemic heart disease [22]. Although this study used proxy measures like tooth loss for periodontitis another recent study involving 60,174 participants in the Netherlands clinically diagnosed periodontitis and found that it had an independent association with atherosclerotic CVD [23]. Similar associations have been reported in systematic reviews and meta-analyses [1, 17, 24–27].

In light of current evidence there is growing emphasis to include oral health as part of cardiovascular care. Unfortunately international reports suggest that only a few with CVD seek dental care due to lack of dental care access, as well as lack of oral health awareness [28].

Although the effectiveness of periodontal treatment in improving cardiovascular outcomes has not been confirmed [18, 29, 30], there is international consensus that patients with CVD need to be made aware of the importance of oral health and recommended that a risk assessment and referral to see a dentist be provided [8, 14, 31, 32]. A recent scoping review has also shown that nurses who are involved in providing cardiac care could play a more active role in delivering preventative oral health care [33].

There is a paucity of research and strategies focusing on oral health care and CVD [33] and to our knowledge, no study has explored the views of patients with CVD towards oral health in the in-hospital or outpatient cardiac setting. Gathering this information could inform future preventative oral health programs in this area. The aim of this study was to explore the perception of patients with CVD towards oral health and the potential for cardiac care clinicians to promote oral health. The research questions included:

- What are the experiences and behaviours of patients with CVD towards oral health?
- What is the knowledge of patients with CVD about oral health and its impact on cardiovascular outcomes?
- What barriers and facilitators exist for patients with CVD to access oral health services?
- What are current oral health care practices in the cardiac setting?
- What do patients with CVD feel about receiving oral health education, assessment and referral from cardiac care clinicians in the cardiac setting?

## Materials and methods

### Design

This study is part of a larger project seeking to develop a cardiovascular oral health program for cardiac care clinicians to provide oral health assessment, education, and referrals for patients with CVD. As an initial step, a needs assessment was undertaken to explore the perceptions of patients with CVD regarding oral health. A qualitative approach using semi-structured interviews was utilised to answer the research questions.

### Sampling and setting

Purposive sampling was used for the selection of study participants. Flyers advertising the study were distributed among cardiac rehabilitation units at three large metropolitan hospitals (Liverpool, Fairfield and Balmain) in Sydney, Australia. Nurses working in these units also distributed the flyers and informed participants about the study during their cardiac rehabilitation recovery.

Interested participants were invited to take part in face-to-face semi-structured interviews. Patients with CVD who were <18 years of age or had a mental or intellectual disability were excluded from the study. The study information sheet was given to interested participants and written consent forms were obtained together with contact details and preferred times to conduct the interviews. Ethics approval for the study was obtained from the South Western Sydney Local Health District Ethics Committee; reference number HREC/15/LPOOL/410.

### Data collection

The semi-structured interviews were conducted between December 2015 and January 2016 at a place and time that was mutually suitable for participants and interviewer. Interviews were audio-taped and lasted between 30 and 45 minutes. A professional healthcare interpreter was used for participants who did not speak English. The interviews were conducted by an experienced researcher and an interview guide was used (S1 Text). At the beginning of the interview participants were allowed to ask questions and were asked to provide brief demographic details. Main topics explored included: perceptions and the importance of oral health, oral health status and behaviours of patients, barriers and facilitators for patients in oral health care, current oral health care practices in the cardiac setting and the acceptability and credibility of cardiac nurses engaging in promoting oral health. Data collection was undertaken until data saturation was reached [34].

### Data analysis

Interviews were recorded, professionally transcribed (and where necessary, translated) and thematically analysed using QSR International’s NVivo 11 software to facilitate coding and categorising sections of the text [35–37]. The main researcher, a doctoral student with extensive clinical and research experience, and a senior research investigator independently analysed and coded the transcripts and through consensus a list of themes, subthemes including relevant quotes from participants was developed. Pseudonyms were used when presenting quotes from participants. Transcripts and demographic data have been provided as supporting files (S2 Text, S1 Dataset).

## Results

### Demographic characteristics

Twelve participants took part in the interviews. Their mean age was 60.33 years (*SD*: 12.49) ranging from 39 to 78 years. Ten (83%) participants were male and just over half were born overseas. Most participants (*n* = 11) were diagnosed as having Ischaemic Heart Disease as per the World Health Organisation classification [38] and were attending cardiac rehabilitation (a 6–8 week exercise and education program in the post-acute phase of their cardiac condition or after discharge from hospital). The main reason for hospitalisation was surgical intervention which included coronary artery bypass graft, cardiac stent, aortic aneurysm repair and valve replacement. Two participants were last admitted for medical reasons involving end-stage cardiomyopathy and a myocardial infarction. Seven (58%) of the participants were from low to middle income families ($40,000-$80,000) while four (30%) were from high income families (>$120,000). Eight participants (67%) were not working at the time of the interview, and seven (58%) had a level of education equivalent to year 12 or less. The majority of participants (*n* = 9) were receiving government health support and less than half (42%) had private health insurance (Table 1).

**Table 1:** Socio demographic characteristics of participants (*n* = 12).

Characteristic	Frequency
Age years, Mean (SD)	60.3 (12.49)
Gender (Male), n,%	10 (83.3)
Country of birth, n,%	
Australia	5 (41.7)
Overseas	7 (58.3)
Cardiac diagnosis (as per ICD-10 [38]), n,%	
Ischaemic Heart Disease	11 (91.7)
Cardiomyopathy (Heart Failure)	1 (8.3)
Diagnosed in the last year, n,%	9 (75.0)
Reason for hospitalisation, n,%	
Coronary artery bypass graft	6 (50.0)
Cardiac stent(s)	2 (16.7)
Aortic aneurysm repair	1 (8.3)
Valve replacement	1 (8.3)
End stage cardiomyopathy	1 (8.3)
Myocardial infarction	1 (8.3)
Main language spoken, n,%	
English	8 (66.7)
Other	4 (33.3)
Combine annual income, n,%	
< $40,000	5 (41.7)
$40,000-$80,000	2 (16.7)
>$120,000	4 (33.3)
Preferred not to answer	1 (8.3)
Not employed, n,%	8 (66.7)
Highest education, n,%	
Year 12 or less	7 (58.3)
Tertiary education	5 (8.3)
Has private health insurance, n,%	5 (41.7)
Has government health support, n,%	9 (75.0)

The main themes that emerged from the interviews were focused on the participant’s experience regarding oral health and CVD. This included their oral health status, behaviour and current knowledge, barriers and facilitators maintaining their oral health, their perception about oral health practices, and the role of cardiac care clinicians in promoting oral health in the cardiac setting (Table 2).

**Table 2:** Main themes and sub themes from the interviews.

**Main Themes**	**Sub Themes**
**Experience regarding oral health and CVD**	
	• Oral health status and behaviour
	• Current knowledge
**Maintaining oral health**	
	• Barriers
	• Facilitators
**Oral health care in the cardiac setting**	
	• Current practices
	• Promoting oral health
	• Role of cardiac care clinicians

### Experience regarding oral health and CVD

#### Oral health status and behaviour

Six (50%) of the participants reported having dental problems varying from bad breath, tooth ache, swollen gums: *“…it can get swollen at times”* (Janette, female, 78 years), and loose or painful teeth. These participants were low to middle income families. Some participants stated:

*“…the gums have receded lower*. *They've opened up some of the lower part of the teeth”* (Mike, male, 68 years).

Four (30%) participants reported that their dental problems impacted on their ability to eat: *“Yes*, *I eat on the other side”* (Allan, male, 71 years), and personal relationships:

*“I'll walk up to my wife and she'll say oh get away from me your breath stinks … Obviously I must have something else that needs looking at”* (Andrew, male, 65 years).

Eight (67%) of the participants had seen a dentist in the last 12 months while two had not seen a dentist for more than 7 years and one participant with full dentures had not seen a dentist for 20 years: *“The only reason I saw a dentist after I had my teeth out was to get replacement teeth”* (Janette, female, 78 years).

Nine (75%) participants did not see the need to visit the dentist if there was no dental problem: *“Until I get a toothache I don't worry about it*. *Well I didn't see one for 49 years”* (Tony, male, 49 years), or if they have no natural teeth:

*“The only reason I saw a dentist after I had my teeth out was to get replacement teeth*. *So there was no other reason for me to see him and that would have been about 20 years ago”* (Janette, female, 78 years)

Oral hygiene behaviours varied across participants. Although most participants said they brushed their teeth daily (*n =* 10, 83%) there were a couple (*n* = 2) that were less attentive: *“when I'm in the shower…at least every second day”* (Tony, male, 49 years). Five (42%) participants flossed infrequently: *“When I can remember I floss”* (William, male, 39 years), while another participant engaged in more aggressive measures to maintain their oral health: *“Sometimes I treat my teeth with oxygenated water but with low volume…diluted*. *Rinse them”* (Julio, male, 63 years).

#### Current knowledge

Seven (58%) participants were unaware of the relationship between oral health and CVD: *“Never heard of oral health with being a relationship to heart—it's never been discussed with me”* (Mike, male, 68 years), and did not think that poor oral health could affect cardiac outcomes: *“I wouldn't think so”* (Andrew, male, 65 years). Five participants (42%) had a general idea about oral health and CVD but were unsure of the details:

*“When I go to my local dentist he does have sign up in his reception saying that there is a link*. *I didn’t understand the details”* (Steve, male, 51 years).

The main source of information for these participants was the dental professional:

*“They told me that the oral health or the health of the teeth affects the internal organs…*.*That is what the dental nurse explained to me”* (Danny, male, 71 years).

*“This dentist told me that studies found that there is some linkage with heart attack when you teeth are not clean…”* (Lisa, female, 48 years).

However, two (17%) participants who had poor oral health prior to being diagnosed with a cardiac condition reported receiving conflicting information from dental professionals:

*“I told her* [dentist] *that I had a stroke and heart attack and she told me no*, *that one thing* [oral health] *had nothing to do with the other”* (Julio, male, 63 years).

All participants were keen to receive further oral health information and often tried to source it on their own as no information was provided in this area: *“No information packs or anything*, *just Google”* (William, male, 39 years).

### Maintaining oral health

#### Barriers

Nine (75%) participants identified cost as a major barrier to access dental care:

*“I think it's just more the obstacle in regards to the finance*. *I don't see many people wanting to go and see a dentist and pay* [AU] *$250*. *I just don't see it logical for someone and then their rent is $500 a week”* (William, male, 39 years).

*“It is firstly expensive*, *it cost me money it is private and did not want to do it*. *I don’t have extra a few hundred dollars to do this thing”* (Lisa, female, 48 years).

Other issues included difficulties making an appointment such as waiting on the phone for a long time to get through to the public dental service:

*“When I call they do not respond immediately and it takes time*. *I called from home and I was not successful*. *…She* [rehabilitation nurse] *then told me that at the next exercise day she was going to call* [the public dental service] *and wait while my husband was exercising*. *She put the phone on speaker and she called*, *she called*, *she called*, *she called…until they answered”* (Wife of participant–Julio, male, 63 years).

Some participants identified not being aware of a public dental service. Four (33%) participants, in view of their cardiac problem, did not consider oral health a priority as a participant stated: *“I've got heart failure; I don’t think rotten teeth or a sore tooth is going to be anything of a hassle for me”* (William, male, 39 years).

Lack of awareness was another identified barrier (*n* = 5, 42%):

*“I'd never heard of it before…I've still got all my teeth so my attitude was why I should go to a dentist*?*”* (Tony, male, 49 years).

Lack of transport was a problem to some (*n =* 3, 25%) of the participants:

*“Because I am not driving*, *I don’t know my way around very well*. *I have issues with transport”* (Danny, male, 71 years).

One participant was concerned about his lack of mobility and attending dental care:

*“Well I haven't been to the dentist since I've lost the leg*. *So I don't know what the challenge is going to be to get in there and then get into his chair”* (Mike, male, 68 years).

Five (42%) participants simply did not like visiting the dentist due to previous bad experiences including pain or discomfort associated with dental treatment, long waiting times and managing multiple medical appointments:

*“…waiting*, *pain*. *Look*, *between us I don’t like sitting and waiting for a doctor*, *any doctor… waiting one hour*, *half hour*. *I have three doctors*: *my GP* [general practitioner], *my back specialist*, *my heart specialist… all the time I go to doctors*. *I must visit three doctors so I don’t like to make it four”* (Allan, male, 71 years).

One participant feared that dental treatment could lead to complications as his medication included anticoagulants:

*“I was on blood thinners*, *so I didn't really want him to pull a tooth out while I was on blood thinners”* (Andrew, male, 65 years).

#### Facilitators

Factors that helped participants seek oral health care included receiving financial or other support such as transport or having private health insurance:

*“I’m covered*. *I am in the defence force so all my medical treatment is covered by them so I can get in very easily most times to see a dentist”* (Carl, male, 49 years).

Being independent was a facilitator to access dental care. Social support as well as having facilities with transport was also identified by participants:

*“My wife is a vital part of my present life because she transports me from one place to another and she is my support”* (Julio, male, 63 years).

A male participant talked about having a state concession card for transport which facilitated his medical appointments including visiting a dentist:

*“The bus goes past my door*. *I only have to walk down two houses to get on the bus*, *lets me out the front of the hospital*. *Couldn't be easier*, *it's* [AU] *$2*.*50 and on the one ticket”* (Andrew, male, 65 years)

Awareness and the perceived importance of oral health care were other facilitators for some participants:

*“I’ve been brought up that way; you take proactive care of your teeth…it is part of my lifestyle”* (Steve, male, 51 years).

### Oral health care in the cardiac setting

#### Current practices in the cardiac setting

Nine (75%) participants highlighted that oral health was not discussed during their cardiac care and ten participants (83%) had never received any oral health promotional material from cardiac care providers: *“Absolutely nothing on oral health”* (Janette, female, 78 years).

The only times when oral health was discussed was when it was initiated by the patient:

*“All started when my wife told the rehabilitation nurse about my teeth problem and she gave her the telephone number of the dentist here in the hospital”* (Julio, male, 63 years).

Or if they had diabetes mellitus:

*“I'm a diabetic as well and one of the doctor's said that diabetes and oral health*, *there was a link between them*. *But I don't know anything else”* (Andrew, male, 65 years).

Oral health was also discussed when patients needed valve replacement surgery as they were required to see a dentist as part of current pre-surgical admission protocol (*n =* 2, 17%):

*“…the only thing I came across the link was in the package they gave me before surgery…in particular in regards to valve replacement”* (Carl, male, 49 years).

However, one participant reported not being provided a proper explanation on why he needed to see a dentist.

*“The surgeon's secretary rang me and said the doctor would like you to go see a dentist to make sure there’s no infection in the gums and that… about a week before [cardiac surgery]*. *I was amazed with that*. *I'm on the phone going what*? *Dentist*? *What for*? *You know I've got sore heart not sore teeth*. *I was shocked*. *Yeah*, *I didn't know”* (Tony, male, 49 years).

#### Promoting oral health in the cardiac setting

Eleven (92%) participants thought the best time to talk about oral health was after the acute phase of their condition has passed. Another appropriate time was when discharged from hospital or during cardiac rehabilitation: *“…leaving the hospital we should definitely get something”* (Janette, female, 78 years), *“I think at the start of rehab it will probably be the best time”* (Carl, male, 49 years), *“it is important to get informed during rehabilitation”* (Julio, male, 63 years).

Participants suggested a variety of ways of receiving oral health information from nurses or other cardiac clinicians such as leaflets: *“Leaflets…so I can sit down and have a read of it over a coffee”* (Steve, male, 51 years), in electronic form, via the media or during cardiac education sessions:

*“… in the media in relation to TV we could get an ad out there*. *Because there is nothing telling anyone about this problem that there is a link to the teeth and that causing problems for people with heart disease*. *I think they* [people] *do need to know*, *especially on TV”* (Mike, male, 68 years).

*“Yes*, *because all the talks that we have* [in the cardiac setting] *relate to our cardiac health and this one* [oral health] *doesn’t*, *is missing”* (Janette, female, 78 years)

Two (17%) participants also suggested having information about oral health and CVD at the dentist: *“That could be started at the dental point and carried on throughout”* (Mike, male, 68 years).

One participant commented on how important it was to have oral health awareness:

*“If people don’t understand that if you do not brush your teeth you are going to have a heart attack they will be more motivated*. *I would’ve not done it if my friend did not drag me and force me into seeing the dentist*. *I was probably not going to see him for a long time*. *Now I see that it was very good because my heart probably would have taken longer to heal up if I didn’t clean up properly”* (Lisa, female, 48 years).

#### Role of cardiac care clinicians promoting oral health

All participants were receptive to cardiac nurses promoting oral health in the cardiac setting and were comfortable with the idea of nurses educating, assessing and referring patients to oral health professionals when necessary:

*“I will take advice from someone that's a cardiac nurse in regards to my teeth… I feel comfortable coming to the nurses most definitely”* (William, male, 39 years).

*“…because you're in and out of the hospital with programs and stuff*, *you're quite familiar with some of the nurses*, *so all ears for whatever they have to say”* (wife of participant–William, male, 39 years).

One participant was mainly comfortable with nurses assessing his oral health as long as there were no dental interventions: *“as long as she* [nurse] *doesn't shove things in my mouth that turn around and taste rotten”* (Mike, male, 68 years).

Other cardiac care clinicians identified by participants to have a role promoting oral health were physiotherapists: *“all the nurses and physios that were in rehabilitation are absolutely brilliant; it is fine if they tell me about my oral health”* (Carl, male, 49 years).

However, some participants (*n =* 4) were unsure if they have enough training to promote oral health: *“I don’t know if they are trained enough to explain…”* (Robert, male, 72 years).

## Discussion

The findings from this study show oral health problems are prevalent with half of the participants reporting dental issues at the time of the interview. As well the interviews showed that some of the barriers to access dental care include the financial burden associated with dental visits and lack of oral health awareness or misinformation and difficulty accessing public dental services. Facilitators include having financial and social support. People with CVD are receptive towards receiving oral health information, assessment and referral by cardiac care clinicians in the cardiac setting.

The incidence of oral health problems is not surprising as Australians with lower incomes are more likely to have oral health problems such as untreated tooth decay and missing teeth and the incidence is higher in older adults. Further, according to national statistics more than a third of the Australian population above 45 years suffer moderate to severe periodontal disease [10].

Over half the participants in this study reported seeing a dental professional in the last 12 months, two of which were for mandatory checks prior to having valve replacement surgery. This is consistent with a previous study that reported poor oral health practices among people with heart disease [39]. Although one study reported that only 38% of people with CVD sought dental care [29], no specific Australian data on the oral health status or uptake of dental services is available for patients with CVD. More general data on the oral health status of Australians show that about 44% adults have regular (every 6 to 12 months) dental visits [10].

The financial burden associated with dental visits and treatment was identified by most participants as a major barrier to seeking dental care. Results from the Australian National Dental Telephone Interview Survey report that from 1994 to 2013, there has being a steady increase in the proportion of adults avoiding oral health care visits and following recommended treatment due to cost, even when they have private health insurance [10]. In Australia, the cost of a dental visit can vary greatly from dentist to dentist as regulations via regulatory agencies do not extend to pricing [40, 41]. Exacerbating the problem is the difficulty accessing public dental services as these are only available to people with health care card or pension card holders [42]. In the current study, two participants mentioned having to wait for long periods on the phone to organise an appointment, and another two participants were not aware that they could access the public system for dental care. The issue of disadvantaged people having serious access problems and extensive waiting times to the public dental care system is not new, and has been previously identified in the Australian context [43].

The study findings also indicate a lack of awareness or misinformation regarding oral health and CVD. Even though some participants had some idea of a possible connection between poor oral health and CVD they were unaware of the relevance of this link. Some participants described receiving incorrect information from dental professionals when enquiring about a possible link between their poor oral health and current cardiac problems. Unfortunately there are no comparable studies that have explored the oral health awareness of people with CVD or the knowledge among dental professionals. Looking beyond the CVD setting, many patients in maternity, palliative care, immune-compromised and with diabetes are also unaware of the importance of oral health [44–47]. Perhaps of greater concern are the findings from several studies that reported dentists were also often unaware of the impact of poor oral health on systemic conditions [13, 48–50]. The issues of lack of oral health awareness, access to public dental service and cost highlighted in this study have also been reported as significant barriers for pregnant women seeking dental care [51, 52]. This may indicate that access issues to dental care is not isolated to people with CVD but is also a common problem among other people with other health conditions where poor oral health is associated with negative health outcomes.

This lack of oral health awareness among CVD patients is likely due to the limited amount of dental care information provided by their clinicians. Participants reported that no information about oral health was provided during their cardiac appointments or other healthcare encounters, except the mandatory visit to the dentist in heart valve surgery, as part of current protocols [53]. However, even in these situations patients were not provided with a proper explanation as to why they needed to visit a dentist. This lack of discussion is consistent with findings of a recent scoping review which highlighted a lack of emphasis on oral health by cardiac care clinicians or cardiac health professionals [34].

Facilitators for accessing oral health care identified in the study included having financial support and/or private health insurance. In Australia coverage of dental costs by private health insurance is based on individuals or families purchasing a health insurance policy, which covers all or part of the cost of visiting a private dentist. The Australian national dental telephone interview survey in 2013 reported that people with private health insurance have better oral health, access dental services more often and follow treatment compared to people that attend public oral health services [10].

Social support was identified as an important component to people accessing medical care especially among those requiring instrumental support during the acute, recovery or maintenance phase of their condition [54], for example, a spouse to provide transport to all health-related appointments.

This study has also for the first time explored the perceptions of people with CVD towards oral health care in the cardiac setting. Most participants preferred to receive oral health education after the acute phase of their condition or on discharge from the hospital or during the cardiac recovery or rehabilitation time [55, 56]. Unfortunately attendance to cardiac rehabilitation is poor with only about 30% uptake on referrals [57] therefore development of an oral health program needs to address this issue to reach the wider cardiac population. Participants voiced that they would like resources and information in a variety of ways including written (leaflets, booklets, posters), electronic (email, internet) or through the media (patient information, advertisement). In Australia, as in other developed countries, almost 60% of adults have low individual health literacy therefore oral health information resources need to be tailored to the intended consumers and be easy to navigate, understand and use [58]. As patients worry about seeing a dentist when they have just started anticoagulant therapy, education about the importance of maintaining their oral health and having regular dental check-ups needs to be addressed.

The potential for cardiac care clinicians to promote oral health was also explored in this study. All participants were receptive to nurses providing education, assessing their oral health and referring them to dental services if necessary. Some commented that they would also be receptive of other cardiac care clinicians such as physiotherapists providing oral health education. Even though the role of nurses or other cardiac care clinicians promoting oral health in the cardiac setting has not been explored in Australia [59] or internationally, the nurses’ role in oral health has been pursued in other settings such as maternity, paediatric and aged care [60–67]. Nurses have a close relationship with cardiac patients in the acute setting for several days, and require assessment by the cardiac nurse prior to discharge. In the outpatient settings, patients who attend phase II cardiac rehabilitation work closely with the nurse(s), physiotherapist(s) or exercise physiologist for at least 6 to 8 weeks; therefore it is not surprising that participants would feel comfortable with cardiac care clinicians promoting oral health. However, some participants were unsure if nurses possess sufficient training to be able to promote oral health therefore this issue would need to be addressed to prepare nurses to undertake this role. In the United States of America (USA) oral health training was incorporated in 2012 as a national initiative in nursing training to improve the quality of oral health and reduce disparities [68]. However, in Australia undergraduate nursing programs do not include oral health training [69]. Other potential barriers that need to be taken into consideration if oral health promotion training is to be provided by nurses may include time constrains in the acute setting [70, 71], low level of confidence [64, 72, 73], and limited knowledge and training [52].

Encouraging cardiac care clinicians to raise oral health awareness has the potential to improve the oral health status and behaviours of patients with CVD. A systematic review of the effectiveness of oral health promotion among patients with CVD found that oral health promotion activities, including raising awareness, seems to improve periodontal health and have a positive effect on the behaviours and knowledge of patients as well as health care staff in the cardiac setting (29). Although improving the oral health of people with CVD may not necessarily reduce cardiovascular outcomes in the long term [14, 30, 31, 74–76] it will reduce the presence of infection and bacterial load in the body which is always beneficial for patients with CVD [31]. In addition, maintaining good oral health will reduce the need for people with CVD, especially those with reduced cardiac functioning, to undergo emergency dental interventions like extractions [77]. These procedures are invasive and can lead to stress and increased circulatory dynamics putting them at higher risk of heart failure [78].

## Conclusions

Findings from this study suggest that many patients with CVD have dental problems yet they lack awareness of the importance of oral health and its potential impact on cardiovascular outcomes. Exacerbating the issue is limited oral health advice being provided in the cardiac setting and difficulty accessing timely and affordable dental services. Cardiac care providers could play an important role in promoting oral health in the post-acute phase provided they are formally trained in this area. Preventative oral health programs need to be developed in the cardiac setting that involve cardiac care providers and address dental referral pathways. Such programs could potentially provide people with CVD in Australia the opportunity to receive appropriate oral health education, assessment and referrals.

### Limitations

This study involves a small number of mostly male participants with CVD attending cardiac rehabilitation in three different hospitals in Sydney Australia therefore results may not be generalisable to other Australians with similar conditions. However a larger quantitative study involving patients with CVD is being undertaken to confirm the results of this study.

## Supporting information

S1 Text Interview guide. (PDF)

S2 Text Interview transcripts. (PDF)

S1 Dataset Demographic data. (XLSX)
