# Supplementary material for: Oral health and cardiovascular care: Perceptions of people with cardiovascular disease
Source: PLoS One. 2017 Jul 20;12(7):e0181189. doi: 10.1371/journal.pone.0181189 (PMC5519046; doi:10.1371/journal.pone.0181189)
Supplement: S1 Text — (PDF) [file pone.0181189.s001.pdf]

Interview guide

Introduce myself. Thank the person for participating in the interview.

The main purpose of the interview is to explore YOUR perceptions about oral health and cardiovascular disease and cardiac nurses providing oral health education, assessment and referrals.

Confirm consent to participate. Explain consent to be audiotaped. Participation is voluntary, confidentiality and anonymity.

Sign and collect consent form.Collect demographic information.

Commence audio recording.

Now I would like to talk about oral health (mouth, teeth and gums) and CVD.

Current knowledge

- Can you tell me what do you know about the topic of oral health (the health of your teeth and gums) and cardiac (heart) disease? Introducing the topic Explore

- Is maintaining oral health in CVD important for you? Elaborate

- Can you tell me if you have ever received any information about oral health since diagnosed with a heart problem?

- Can you tell me what information have you received? Explore what information, when, by whom, in which form, good, bad, elaborate

- If no information received, would you like to get information?

- Can you tell me how would you like to receive information, in which form? (eg leaflet, internet, media, etc)

- Would you like the information in your own language or in English? (NESB patients)

Needs & Practices

Now we just want to talk about your dental health needs

- Have you got any current concerns about the health of your mouth, teeth and gums? Elaborate

- When was the last time you saw a dentist?

- What oral health practices do you follow? What do you do to look after your oral health? Elaborate

• Tell me about your experience going to the dentist or dental service. (when did you go? What happened? Did you need treatment, explain. Elaborate

- Do you face any challenges to look after your oral health or see a dentist (dental professional)? What are those challenges? Elaborate

Barriers and facilitators

• What are your views about including oral health information when attending cardiac rehabilitation?

• What are your views about having an oral assessment when attending cardiac rehabilitation?

- What do you feel about responding questions about your oral health? What about having a visual inspection?

• What do you think or would you be comfortable reciving information and having an oral health assessment by the cardiac rehabilitation nurse? Elaborate

• When do you think is the best time to receive this information?

- Can you tell me what sort of support do you need to be able to look after the health of your teeth, mouth and gums?

Referral

• Would you follow the nurses' advice about your oral health, for example get referred to see a dentist? Why or why not?

- Do you think nurses have enough knowledge to provide information about oral health?

Social support

• Can you tell me about your social support (who supports you, living arrangements, family/ friends/ health or other services to attend appointments and other commitments? What type of trasport do you use?.

• Are there any other comments or suggestions you would like to make about this program or any other issues?

Thank you again about participating in this interview
