# Supplementary material for: Oral health and cardiovascular care: Perceptions of people with cardiovascular disease
Source: PLoS One. 2017 Jul 20;12(7):e0181189. doi: 10.1371/journal.pone.0181189 (PMC5519046; doi:10.1371/journal.pone.0181189)
Supplement: S2 Text — (PDF) [file pone.0181189.s002.pdf]

Interview 1

FILE DETAILS

Audio Length: *19 minutes*

Audio Quality: *High*

Number of Facilitators: *One*

Number of Interviewees: *Two*

Difficult Interviewee Accents: ☐ *Yes* ☒ *No*

Other Comments: Facilitator interviewed in Spanish

Interviewer: I just want to confirm that you are happy to record this interview?

Patient: Yes...yes

Interviewer: You mentioned that you had a heart attack and a stroke, how long ago was that?

Patient: In April last year, 2014, I had a stroke and about 3 months ago the heart attack in September

Interviewer: What do you know about oral health and the heart? Have you ever heard anything or have any idea about the subject?

Patient: I have no idea.. I haven't got a minimal idea about what happens but I have the impression that they are things that are together because I had the stroke and after I had the heart attack and then all my teeth got loose and I started having dental problems

Interviewer: Ok, so you thing that there is a relation between the oral health and the heart..

Patient: Very possible, very possible…

Interviewer: What about people that already have cardiac problems, is there a relationship there?

Patient: To be honest I haven't ask. I first thought that it was due to some medicines or tablets we are given in this situation (heart problems) I believed it before but not now. The dentist told me that it is not related

Interviewer: Did you ask the dentist?

Patient: Yes I did

Interviewer: How long have you been attending the rehabilitation program?

Patient: About 6 o 7 months in this program

Interviewer: Do you think that if a person has oral or dental problems it can affect the heart?

Patient: No, I do not think so

Patient's wife: I do, I think so

Interviewer: How important is for you to maintain your oral health?

Patient: It is extremely important. Let me tell you that for me I never liked my teeth since I was a child and I think that this affected my personality, my self-esteem and all that in front of other people. Even now you do not want to show your teeth, I sort of feel embarrassed

Patient's wife: He cannot eat well…cannot eat well

Interviewer: And eating is there a problem?

Patient: Also, cannot eat well

Interviewer: Did you have any dental problem before your diagnosis

Patient: No

Interviewer: So, after you developed heart problems and the stroke you starting having issues

Patient: Yes

Interviewer: Since you have been diagnosed with a heart problem has anyone told you anything about oral health or given you any information? Have you heard anything about it?

Patient: No, nobody have told me anything

Interviewer: Nothing written, television, verbal?

Patient: No, nothing

Interviewer: Would you like to receive information? Do you think it is important?

Patient: Of course is very important, we need to be informed. Furthermore I think it is important to receive information before you have a heart attack because many people are at risk or having a heart attack, don't you think? Until now I didn't know anything and if there is any relationship people should be informed I believe

Interviewer: When I saw you last Monday you told me that the rehabilitation nurse have just organised you an appointment to see the dentist. Can you tell me about that and how did it happen?

Patient: All started when my wife told her [rehabilitation nurse name omitted] about my teeth problem and she gave her the telephone number of the dentist here in the hospital. She told my wife that to make an appointment she needed to be patient as it takes time to get an appointment date.

Patient's wife: Yes, yes I spoke to her, I asked her if there was a dentist here in the hospital, she told me yes, gave me the number and told me to be patient when I call as they do not respond immediately and it takes time. I called from home and I was not successful. Then one day she [the rehabilitation nurse] was here at the rehabilitation program during exercise and asked me how we went at the dentist, I told her that bad as we did not get any response. She then told me that at the next exercise day she was going to call and wait while my husband was exercising. She put the phone on speaker and she call, she call, she call, she call…until they answered.

Interviewer: Did she get an appointment? Was it quicker?

Patient's wife: Yes! She called in Thursday and he had the appointment for Monday

Patient: Yes, it was very quick

Interviewer: But when you called you weren't successful…

Patient: No

Patient's wife: But I could not be calling and calling from home…

Patient: The lady at the dentist told us we were lucky because we got the appointment very quick that should have been taken about 3 months…

Interviewer: In relation to your oral health, how do you feel now? Do you have any needs or problems?

Patient: What I have is an emergency. I cannot eat well, if I want to eat meat I have to be careful because I can lose my teeth as they are all loose. The doctor told me that it is possible that at my next visit I will have more missing teeth.

Interviewer: Oh…and when you went to the dentist were you offered any treatment? Are they going to give you any treatment?

Patient: Yes I will have treatment

Interviewer: What type of treatment?

Patient: They going to put false teeth, dentures

Interviewer: And how do you feel about that?

Patient: Good, good…until I have them I feel good

Interviewer: Do you think it will be good having dentures?

Patient: I know that is not good

Interviewer: Not good?

Patient: No, is not very good because now there are many more advances in dentistry. They can put many other things that are better than dentures, but is very expensive I know..

Interviewer: So, to confirm the information you gave me. When was the last time you went to the dentist?

Patient: Last Monday

Interviewer: Let's talk about your habits. What do you do to look after your teeth?

Patient: I wash my teeth one a day. Sometimes I treat my teeth with oxygenated water but with low volume…diluted. Rinse them

Interviewer: Since when you have noticed your teeth were loose?

Patient: Since the stroke, last year

Interviewer: Tell me, how was your experience with the dentist?

Patient: It was good, very good. Because she told me immediately what I had, she took x-rays and told me what I had. I knew what I had

Interviewer: Did she give you the name of what you have or only told you that your teeth were loose?

Patient: No, she told me I have an infection in the gum and that got my teeth loose.

Interviewer: Did she tell you that it was related to anything?

Patient: No. I told her that I had a stroke and heart attack and she told me no, that one thing had nothing to do with the other

Interviewer: Do you have any problems seeing the dentist regularly?

Patient: No

Interviewer: Do you get scared?

Patient: Yes, I get scared. If I see the syringe twice I want to run away. That day I did not run because I pretended to be macho…

Interviewer: Talking about information about oral health, what do you think about including oral health information during cardiac rehabilitation?

Patient: I think that it is very important to all people who had a heart attack, it is important to get informed during rehabilitation

Interviewer: And what do you think about being asked about your oral health by the nurse?

Patient: I think that all people would be comfortable. I think that oral health is something private for the person and we do not want to talk about it, nor many people want to talk about it, you keep it. Therefore is important to say something and others ask so we can get help.

Interviewer: So, how would you feel with the nurse having a look at your mouth?

Patient: I feel ok with that. In the last years I have spent so much time in hospitals so it was like a therapy if the nurse checks here or there doesn’t matter is all for my heath so is ok.

Interviewer: When do you think is the best time to receive information or being assessed?

Patient: During rehabilitation I think is the best time to be assessed and reviewed

Interviewer: And how would you like that information delivered?

Patient: Like what you are doing now interviewing people when they are finishing their rehabilitation. Also written like a leaflet.

Interviewer: If the nurse gives you advice to go to the dentist would you do it?

Patient: Yes

Interviewer: Do you think that nurses are sufficiently prepared or trained to provide information in this subject?

Patient: I think so. There are nurses that know a lot, they are very advanced

Interviewer: So you feel comfortable with nurses giving you information. Are you confortable asking them questions?

Patient: Yes

Interviewer: I have noticed that your wife is always with you. Is she your major support? Do you drive?

Patient: Yes, my wife is a vital part of my present life because she transports me from one place to another and she is my support.

Interviewer: Any other family members support you?

Patient: No, just her. She takes me to appointments, in the hospital when I was sick it was her

Interviewer: Do you need any extra support at home for you activities of daily leaving? Has anything change since before?

Patient: No, is all the same at home

Patient’s wife: Before he got his own clothes. Now I have to do it as sometimes he puts the clothes back to front…he needs help with that. But slowly, slowly things are getting better…

Interviewer: If a regular free dental service is offered to you and cardiac patients, would you attend it?

Patient: Exactly, of course

Interviewer: Have you got any comments to add?

Patient: A comment? As you are organising this there should a program call “The smile of (Name omitted)” so all the older people that smile with beautiful teeth.

Interviewer: That is nice! Very good idea!

Thank you very much for participating in this interview and thank to you as well [patient's wife] for participating as the information your provided is very important to complement your husband's information. You are an integral part of his heath. Thank you again.

Interview 2

FILE DETAILS

Audio Length: *41 minutes*

Audio Quality: *High*

Number of Facilitators: *One*

Number of Interviewees: *One*

Difficult Interviewee Accents: ☐ *Yes* ☒ *No*

Other Comments: Interview with a Vietnamese interpreter

Interviewer: Again, thank you for agreeing to participate in this interview

Patient: Yes

Interviewer: As explained before this study is about looking at your opinion and perceptions about oral heart and cardiac conditions

Patient: yes

Interviewer: and also about your opinion about nurses giving you information and referring you to the dentist if necessary

Patient: For the dentist I go to a dentist to do a clean-up once a year and if you refer me to an extra dentist I am happy to go to that extra dentist

Interviewer: At the moment we are looking at the patient's opinions about developing a program, hopefully we will be able to do it in a couple of yet, not yet. In the study we are looking at different people's opinions patients and nurses…

I wanted to ask you, how long have you being coming to the exercise program?

Patient: Since November until now, probably 5 to 6 weeks

Interviewer: Have you attended another program like this before?

Patient: No

Interviewer: What do you know about heart problems and the mouth, oral health? Have you heard anything about it before?

Patient: No, I haven't heard

Interviewer: And of the relationship between heart and mouth? Any links?

Patient: When I went to the dentist to clean-up they told me that the oral health or the health of the teeth affects the internal organs. Besides that I am diabetic so it is related to teeth and my heart. That is what the dental nurse explained to me when I went to the dentist

Interviewer: So the dental nurse told you that there may be problems with the heart?

Patient: Yes, and because they explained it like this, every night I brush my teeth properly and floss as well so it won't be an infection so it is good for my health. So because the nurse told me I have being following that up for a few years so my health is good besides I do not have bad breath…

Interviewer: So when was this? How long ago the nurse told you this?

Patient: About 2 years now. Because the government had a subsidy for people who are diabetic I understand that it was roughly 2 thousand dollars so I went to the doctor and to the dentist

Interviewer: That right I remember that it was about 2 or 3 years ago

Patient: Yes, they recently cut this program this year or the year before…

Interviewer: So because of that you now know that oral health can affect the heart?

Patient: Yes

Interviewer: That's good, very good… How often do you go to the dentist? When was the last time?

Patient: I know the dentist for more than 10 years so every now and then if I haven't gone for a while he will ask the front counter staff to contact me

Interviewer: So, when was the last time?

Patient: Beginning of 2014

Interviewer: When the nurse gave you information about the diabetes, the mouth and the heart, did she give it to you verbally or in written form?

Patient: Verbally. Because there are people that have worse situation that me, the dental nurse just told me verbally

Interviewer: Would you like to receive more information?

Patient: If you have some information, I will take it home to let my children explain to me

Interviewer: If there was information, how would you like it given to you? What do you prefer?

Patient: I would like a leaflet or something like that so I can take home to my children. Because I don't know the language so I have to rely on my children…

Interviewer: But if it was written in Vietnamese or Mandarin, would you prefer that?

Patient: Please give it to me in English because my children have education, they can understand and they can explain to me

Interviewer: No problem. This is why we are doing this interview so we ask people they preference and we can provide information that does not exist in Australia and also train the nurses.

Patient: Thanks to you because if you have information to give to people for example future patients you can develop a program to help people in the future.

Interviewer: Thank you, thank you

Patient: Now I would like to talk about your mouth and your teeth, have you got any problems that you worry about at the moment?

Interviewer: No

Patient: What do you to look after your mouth? You mentioned that you brush your teeth and you floss, how often do you do that, how many times per day?

Interviewer: Morning and night

Patient: That's good.

Interviewer: I use the dental floss, the doctor told me to use the one like a hook with the floss in the middle, it is better than the floss. The dental nurse told me…

Patient: When you went to the dentist the last time, did you need any treatment? Did they need to do anything with your teeth or mouth?

Interviewer: I had filling done and clean-up. They put something in my teeth. The procedure lasted about 2 hours. They put something to strengthen the teeth or something like that…

Patient: No, no. Last time I see the dentist not do filling...Because I had 2 teeth that were not very strong, a bit wobbly, they told me that they need to be extracted so I need to cleaned up, she instructed me what to do so I followed her instructions at home and I think now they are stronger.

Interviewer: That's very good. Now, do you see any challenges seeing the dentist or looking after your mouth and teeth?

Patient: No, no problems, no difficulties. The only thing is that I have to see the dentist every year and now the government doesn't subsides it so I have to pay with my own pocket money, around 300 or 400 dollars each time.

Interviewer: so do you make your own appointment?

Patient: The dentist makes appointment, first was every 6 months and then every year and now it's been a year but they haven't call me

Interviewer: I understand. It is expensive, isn't it, you have to pay

Patient: Yes, because I don't have private health insurance the doctor has to see what has to be done…for example if I have private health insurance they would be prepared to pay for support how many thousands a year and then the doctor would follow that and see what procedure needs to be done and see how much the health insurance would pay and then claim from that but because I don't have health insurance…

[Interruption. Patient had to attend the exercise program]

Interviewer: Going back to the questions I was asking before, how do you feel about getting information about oral health during rehabilitation program?

Patient: Because I do exercise I feel my health has improved. I don't feel tired anymore, I can walk…

Interviewer: Do you think is a good idea to receive information about your teeth and oral health while in the program. With all the information you get in the cardiac rehabilitation program would you like to have this [oral health] information included?

[Clarification of this question with the interpreter]

Patient: Yes I would like to

Interviewer: And when do you think is the best time to receive this information?

Patient: The head nurse during the exercise said that after the exercise is finished she wants to see me and my son about another program…

Interviewer: Do you feel comfortable with the nurse asking you questions about your mouth and teeth and also how do you feel about the nurse looking at your mouth, doing an assessment?

Patient: I think is ok, no worries, no problem

Interviewer: If the nurse gives you advice to see the dentist for example, would you follow that advice?

Patient: Because I am not driving, I don't know my way around very well if I have to have appointment if my children have time is ok but if they go to work I have issues with transport but if the appointment is here at the hospital I can go by bus

Interviewer: Ok. Do you feel that the nurses are trained enough to give information about oral health?

Patient: It is their responsibility. Because, if I have problem I asked them, for example you know if food affect the heart and what sort of diet it is I can ask them at the appointment time through the interpreter..

Interviewer: Yes, so he feels that the nurse is prepared enough to give that information?

Patient: mmh..See, the hospital nurse I don't know whether they have enough information like to guide me with those things but that is their area of practice for me…because I am limited, because you want to know more I was prepared to participate in your interview.

About nurses and what they do is their area of knowledge I don't know if they are trained enough to explain…

Interviewer: If there is a dental service offered to cardiac patients like you would you go if it is given in the hospital free of charge?

Patient: Yes, agree. Yes, for example last month they had the talk about the heart and the doctors and nurses were there to explain around 10 o'clock to 2 o'clock but I don't know if they have a program next month.

Interviewer: They are doing the program again this Wednesday. They doing the talk in here

Interviewer: I notice that you come to your appointments with your son; do you always go with your children? Do you go by car or other way?

Patient: Yes, on Wednesdays my son is working. If it falls into the time my daughter has to go to work then I will take the bus

Interviewer: Ok, thank you. Is there anything you would like to add or say?

Patient: I thank you very much to have the appointment with you and to participate in your interview I appreciate it

Interviewer: We will take into account your comments to develop this program so it will be easier for patients to go to the dentist

Patient: I do not have any comments but you are responsible to help future patients and it is good to have such a thing for the committee to help the general humanity

Interviewer: Thank you and have a good evening

Interview 3

FILE DETAILS

Audio Length: *29 minutes*

Audio Quality: *High*

Number of Facilitators: *One*

Number of Interviewees: *One*

Difficult Interviewee Accents: ☐ *Yes* ☒ *No*

Other Comments: Interview with a Vietnamese/Mandarin interpreter

---

Interviewer: You are attending the cardiac program right now. Is this the first time you attend a program like that?

Patient: First time

Interviewer: It is about 6 weeks isn't it?

Patient: Yes it is about 6 weeks but if I can continue I will continue…this is good because if you do it outside you have to pay

Interviewer: Have you ever heard anything about your oral health, your mouth and teeth, in relation to your heart?

Patient: No

Interviewer: So, do you know if there is any relationship between your mouth and your heart?

Patient: No I didn't heard, I didn't feel either. I already know that if you eat too much fat too much sugar it is awful for your internal organs

Interviewer: Yes that's right, but what about just your mouth, your teeth, your oral health?

Patient: I never heard of that because I work and then I…if we have a program, an advice program or something then I would know

Interviewer: Yes and that's why we are doing this. We feel that many people don't know and there is not much information given to patients. That is why we are doing this, we are trying to see what the patients need and give them that information

Patient: Yes, last week they had a program talking about the heart, about how to prevent...and diet but if you are not exposed you don't know

Interviewer: They did not talk about your oral health?

Patient: No, only the heart what to avoid…

Interviewer: How important is your oral health for you?

Patient: I don't know I don't understand. I know to protect your teeth and not to have fillings…that's all I know

Interviewer: So you look after your mouth. How often and how do you do it?

Patient: The dentist is the one that looks after our teeth but there are lots of things that I don't know.

Interviewer: Do you brush your teeth often?

Patient: Morning and night

Interviewer: Anything else you do with your mouth?

Patient: No

Interviewer: So, has the dentist or dental nurse told you anything about looking after your mouth or anything else? Have they told you if your oral health affects anything else in your body?

Patient: No, they don’t talk. They just do it and they get the money! They don’t explain

Interviewer: I understand. You said you haven’t received any information about your oral health, would you like to get some information?

Patient: Yes, if they give me a booklet or something to have a look is good

Interviewer: Do you prefer a booklet or leaflet?

Patient: Yes. If you have it is good so I can learn more. You know, the dentists don’t give you information, you go in there, they do they job they get pay…If you have, it is good

Interviewer: Let’s talk about your oral health. Do you have any problems or worries about your mouth or teeth at the moment or its all ok?

Patient: I have 2 bridges one upper and lower

Interviewer: Is it all ok at the moment?

Patient: No problems

Interviewer: When was the last time you went to see the dentist?

Patient: Long time ago, 2 o 3 years ago because there’s nothing wrong, nothing broken

Interviewer: So, you go to the dentist when there is something wrong?

Patient: If there is something wrong other ways I go there and already have to pay over 100 dollars or 200 dollars

Interviewer: Do you go to a private dentist?

Patient: Pay cash…because I don’t pay for private health insurance

Interviewer: And you don’t go through the hospital?

Patient: No

Interviewer: Just private, is it expensive?

Patient: Yes, I have to pay, is not free. I asked before to clean the root, the nerve, the blood vessel in there, I don’t know how to say it…and put something in the top, they said it cost one thousand

Interviewer: I think is called root canal. Last time you went to the dentist they did the bridge?

Patient: Yes last time. Till now nothing…over 2 years…I think is better let them see every now and then not too often…

Interviewer: Do you see any problems seeing the dentist? You already mentioned that is expensive. If you need to see a dentist regularly every few months or every year, do you see that as a problem, what problems do you see?

Patient: I think if there are no problems with my teeth I don’t need to see them

Interviewer: Talking about the cardiac rehabilitation program, would you like to receive information about oral health and how it can affect the body through the program?

Patient: I think if they give me such information is good so I can have prevention or something

Interviewer: Are you comfortable with the cardiac nurse giving you that information?

Patient: Yes, if there is such a program I would like to know more.

Interviewer: What about other cardiac nurses either in the ward after the surgery or in rehabilitation. Are you comfortable with nurses giving you information?

Patient: That's good. If the people here don't tell me or the dentist doesn't inform me of the relationship between oral health and the heart I don't know

Interviewer: That right you don't know. What about the nurses asking you questions and having a look at your mouth, do you feel comfortable with the nurse doing that?

Patient: If it doesn't affect me it is ok for them to check

Interviewer: And ask questions as well?

Patient: There is no problem

Interviewer: When do you think is the best time if you are going to receive any information written or verbal. When do you think patients receive that information?

Patient: If you are not given the booklet how would I know?

[Clarified the question to the interpreter]

Interviewer: When is the best time to talk about it? If there is a leaflet, when would you like to get it: at rehabilitation time or in hospital, when is best to get it?

Patient: If you give it here is convenient…in rehabilitation. It is the first time I heard that the oral health can affect the heart, is the first time I heard…

Interviewer: That is why we are doing this program…

Patient: How does it affect the heart, can you tell me?

Interviewer: I can do that, when we finish the interview I will tell you all about it

Interviewer: If the nurse tells you to go to the dentist and give you a referral would you follow that advice?

Patient: Does it mean that the hospital will refer me or I need to go by myself?

Interviewer: No, the hospital would refer you

Patient: So they will check that the teeth affect my heart, is it?

Interviewer: They will check your teeth and if they think there is a problem would you be happy for the nurse to give you a referral either to the hospital or if you prefer you can go to your private dentist. The idea is that if there was a free dental service and the nurse refer you, would you follow that advice of the nurse?

Patient: This isn't very important, this is not very important to me. You see I am [unclear] if you refer me to the dentist, is that going to allow me to live another 10 or 20 years more or not?

Interviewer: Ok, I'm going to tell him about that

Interviewer: Do you think nurses are trained enough or know enough to give information or teach about oral health to you?

Patient: If you explain it to me I get it but if you want to test me I don't think is necessary

Interviewer: Let's talk about your social support I notice that you come by yourself. If you need to go to an appointment do you go by yourself by train by taxi by car? Who helps you with that?

Patient: Nobody is taking me because they all work. I am still ok to go by myself. I have my family here I have my wife I have my children they are working. My wife ask me if I can go but I say is ok I can go by myself…because I don't want to bother people because they have work and at home you know…

Interviewer: When you had the surgery, did you have somebody helping you at home?

Patient: Yes, my wife. Because I have no son I have to do everything…so after I got out of hospital after a few weeks I mowed the lawn everything…is the same as exercise

Interviewer: You are right is like exercise.

Interviewer: Now if the hospital gives you the opportunity to go to the dentist regularly for free would you go?

Patient: Is it important?

Interviewer: Yes it is. I will explain after the next question.

Patient: If it is free you just go…

Interviewer: I will explain about the relationship between oral health and the heart after. Do you have any comments or anything you want to say?

Patient: No, is ok. I want to know of the relationship…it is related to your health isn't it?

Interviewer: I'm sorry I didn't explain before. With the interview this is what we want to find out from patients, how much do they know and what happens is that not many people know. I am happy to explain now because we have already finished the interview.

Patient: People outside don't know either

Interviewer: I am going to finish with the recording now and I will explain. Thank you for the interview

Patient: I my life can benefit you is good because sometimes I don't know what to say

Interviewer: I understand, you did very well there is no right or wrong, it wasn't too difficult, you answered everything. Now I'm going to explain I will stop the recording. Thank you very much for the interview.

Patient: Thank you

Interview 4

FILE DETAILS

Audio Length: *29 minutes*

Audio Quality: ☒ *High* ☐ *Average* ☐ *Low*

Number of Facilitators: *One*

Number of Interviewees: *One*

Difficult Interviewee Accents: ☐ *Yes* ☒ *No*

Other Comments:

[Aside discussion]

Facilitator: So basically you mentioned that your cardio problems are multiple. You've had a bypass...

Interviewee: I've had a quadruple bypass.

Facilitator: How long ago was that?

Interviewee: That was done in February 2012. Since then I've had another 12, 15 heart attacks and a bucket load of more stenting and ballooning. It started out I only had two stents in there. Within five years I've got a total of about 17 stents and about 14 - no it wouldn't be that many - it would be about 12 balloon procedures.

Facilitator: You've been through a lot.

Interviewee: Yeah, the last one which was only about five or six weeks ago was another stent and another ballooning process.

Facilitator: When did you start with your cardiac problems? When was the first - was it when you did the bypass or before that?

Interviewee: No, no the cardiac problems started back in 1985. I was taken to (Hospital Name Omitted) with a chronic heart problem. They did a couple of things with injections and that. The heart came back to normal rhythm. They just said take it easy; you'll be right. Then when I got to (Hospital Name Omitted) up at the top of the mountain I had chest pain. I was rushed over to the (Hospital Name Omitted). The doctor over there turned around and said I'm not touching him. I'm sick and tired of this. He's got three months. He gets out or I'll put him out.

So I discharged from the air force. Which is something I didn't want to do; I wanted to stay in. But this turkey - he'd never seen me before. He just turned around and said I'm sick and tired of treating him. I'm not going to do a damn thing. He turned around and walked away. This was with chest pain. So I don't think much of him as a doctor.

Facilitator: No I wouldn't either.

Interviewee: So since I've been out - I was going alright for a while. Then approximately 2000, 2001 I started with the chest pains again. I was over at (Suburb Name Omitted) at that stage. I met up with my now wife and moved to the (Suburb Name Omitted) area and had quite a few more. Found a cardiologist to look after me who in turn sent me to the guy that was doing the catheter and then therefore the result of quite a few more stents, balloons and God knows what; and brought it to today's situation.

Facilitator: Through all this - you are attending the rehabilitation program?

Interviewee: Yeah.

Facilitator: Have you attended any programs before?

Interviewee: Yes. I attended back in late 2012 after I had the bypass done. I went to them then and got through the program. I enjoyed doing it as I am now. I still enjoy doing it. The girls are great (Rehabilitation Nurse Name Omitted) is a wonderful woman.

Facilitator: Oh she's beautiful.

Interviewee: The physio that's normally there - I mean there's been so many - now it's (Physiotherapist Name Omitted). She's lovely. You can't get depressed with those girls. They keep you thinking along the lines of hey let's take care of things. That's where things go.

[Aside discussion]

Interviewee: That brings us up to today.

Facilitator: So you've attended twice the program?

Interviewee: Yeah I've done the program once. Now I'm doing it a second time.

Facilitator: This is the six week one isn't it?

Interviewee: This one I'm just going to keep going until I don't need to do it definitely. It's normally a...

Facilitator: It's normally a six or seven week program. This time is six weeks.

Interviewee: Yeah. In my bag I've got another five weeks of home exercise program to carry on. Then on 18 January when I go back I give it to them and they see how the [unclear] been going. They've left it up to me as to whether or not I increase weights or anything like that as I do the exercises or whether I leave it the same. But it's up to me to make the adjustments; which is pretty cool. I found today starting with weights it really did increase the pressure within the exercise. It just showed me also how unfit I am. So I'm looking forward to keeping it up.

Facilitator: Yeah but you have to do it slowly, take it easy.

Interviewee: Oh yeah.

Facilitator: That takes us to the question more specifically about oral health. So from all this experience with cardiac doctors have you ever heard - or even the dentist - have you ever heard of a relationship between oral health and cardiac conditions?

Interviewee: Never heard of oral health with being a relationship to heart - it's never been discussed with me nor has it ever been brought up. It surprised me when (Rehabilitation Nurse Name Omitted) turned around and said there is this link. They would like to interview people. I thought well I've got to get in on this. That's all I need. I don't really need the teeth creating any more heart problems for me.

Facilitator: There is a relationship that we've found. It's been studied more in the last 20 years but there's a very strong relationship that they're finding.

[Aside discussion]

Interviewee: But overall it's quite an interesting fact or interesting subject in that this is taking place. It's serving a different line as to what may be causing some of the heart problems. I was led to believe that you get a heart disease. It's just a disease that keeps playing with the heart and bang it finally closes up. Then I was told that dialysis can cause heart problems. Then I was told that oh yes potassium will create a big problem. If it goes over 6.5 you've got a problem. Well I was really desperate because I was 9.6 - couldn't move. I was as stiff as a board. They took me into hospital and finally brought me back down. But I've been very wary of potassium ever since.

Facilitator: When it's low as well it can cause arrhythmia.

Interviewee: Can it?

Facilitator: So very high or - oh very low yeah. So you have to be careful not to be very high or not to be very low. Keep it balanced.

Interviewee: Okay I'm playing somewhere in the vicinity of 4.8 to 5.8.

Facilitator: For people with renal failure - people tolerate - you can tolerate higher levels than people with normal levels.

Interviewee: That's good.

Facilitator: But it doesn't cause cardiac problems. It causes arrhythmia, so different rhythms in the heart.

Interviewee: Okay.

Facilitator: Anyway I'm back to this - I got myself distracted too.

Interviewee: Yeah I side tracked you.

Facilitator: I love [unclear] as well. So you never heard of the relationship?

Interviewee: No.

Facilitator: Obviously you never had the relationship with people that already have cardiac problems because that's actually the study. They found that people that already have heart problems, when they have an oral problem it can exacerbate that. So it doesn't cause the heart problem. There's no evidence so far.

Interviewee: It just exacerbates it.

Facilitator: But it can exacerbate when they already have problems. Do you feel maintaining oral health is important to you?

Interviewee: Now yes, even more so. I was relatively concerned on oral health because of one - you're talking to people; you don't want bad breath coming out all over them and that. Plus the fact that I'm married to a lovely woman. I don't want to breathe garbage on her. So yeah oral health was quite an interesting point, and a point that had to be done. It was there. But in relation to it being a link to affecting the heart in the way of exacerbating problems that I never knew. So that puts a new standard on making sure that the oral health is even far better.

Facilitator: In fact your oral health habits - what do you normally do for your normal health?

Interviewee: Normally just toothbrush and toothpaste; occasionally some mouth rinse but not very often with the rinse. I think I'll be taking it up as a more regular basis. I've got to find a dentist that will do something about the fact that my gums are receding. The bottom of the teeth are open to the elements. I'm in quite a lot of pain at times.

Facilitator: Are you? So you currently have some...

Interviewee: Oh yeah.

Facilitator: ...have you got any concerns about your...

Interviewee: All the molars are all - the gums have receded lower. They've opened up some of the lower part of the teeth. That's got the open pores going into the nerve and all that. I haven't bought the Sensodyne to try and close it all off. But I think I'm going to.

Facilitator: So are you're saying they've become more sensitive?

Interviewee: Yeah.

Facilitator: The gums are going down into your teeth?

Interviewee: No they're receding back into the gum.

Facilitator: Oh they are receding back - they are exposing. You said it's important to you. You said now it is - now since you've heard from our study...

Interviewee: Yeah.

Facilitator: ....from this study or from before?

Interviewee: It's even far more important.

Facilitator: The next question was in relation to receiving any information but you said you never heard of it.

Interviewee: Never heard of it before until...

Facilitator: Never received any information.

Interviewee: ...I was told [by] yourself.

Facilitator: Obviously if you have the chance to receive information now would you like to get some information about oral health?

Interviewee: Yeah I would, yeah.

Facilitator: How would you like it delivered - verbal, written, television, internet? What would be the best way that it could be delivered?

Interviewee: If we look at the whole thing I think people like myself who are partaking in this interview I think a couple of ways they should be informed (1) Is with the written as a small pamphlet or something like that that's sent out to them. (2) In the media in relation to TV we could get an ad out there. Because there is nothing telling anyone about this problem that there is a link to the teeth and that causing a problem for people with heart disease. I think they do need to know - especially on TV.

Most people that - I'm going to turn around and say most people of the age where it is expected that heart problems would be there; and I'm talking probably 50 up - some of them if they're not working they're just sitting at home watching the box. So it needs to be planted in there. I think that's one of the best ways to do it. But as I say those that have taken part in this shall we say research; I think they should also receive it in a pamphlet form.

Facilitator: Beautiful; that's exactly what we're planning - that's why we're asking questions, to see what you feel. Would you like to receive this information or how do you feel about receiving information through the cardiac rehabilitation program that you attend?

Interviewee: Yeah, not a problem.

Facilitator: How comfortable do you feel about the nurse asking you questions about your oral health?

Interviewee: I doesn't bother me.

Facilitator: Assessing your mouth - looking at your mouth and referring you to a dentist?

Interviewee: It all depends - so long as she doesn't shove things in my mouth that turn around and taste rotten. I'm definitely not going to be in that.

Facilitator: Okay - so you're okay with being given information?

Interviewee: Yeah.

Facilitator: You say you're okay with assessing or looking just a visual, observation.

Interviewee: Paula, I take the attitude and have done for quite some years if somebody is coming in to discuss or have a look and assess or learning. If, shall we say a client meaning myself if they're not prepared to let that person who is learning or trying to discuss things have a go at this, then nobody is going to know for sure. They won't learn properly. In all my times even when I've been in hospital after the heart surgery, the loss of the leg, the whole works I was always asked if they could bring in a trainee and let them have a look; have a go at doing it. I said they've got to learn somewhere. Let's go.

In the finish I ended up doing studies with doctors who were going for their finals exam. They come in and do an assessment and so forth; go out and then front the medical board to find out whether or not they're going to be classified as a full-fledged doctor. [They do].

Facilitator: A lot of patients should be like you.

Interviewee: They get their doctor-ship.

Facilitator: I did ask you about your dental health and how do you feel your teeth are and your mouth is at the moment?

Interviewee: My mouth at the moment I would say would be very poor. The toothpaste I use is nothing fantastic. It's Oral B.

Facilitator: For sensitive teeth?

Interviewee: No just normal. I don't know if there's a better one that I should be using but I'm certainly going out now to find out.

Facilitator: I believe that the ones for sensitive teeth are good for - especially when you're saying that you have a bit of pain because of exposed teeth.

Interviewee: Yeah where the enamel finishes and then you've just got the tooth root is very painful.

Facilitator: When was the last time you saw a dentist?

Interviewee: It would have been late last year. I'm thinking - and it is only going to be a rough stab in the dark - would be about August, September.

Facilitator: Did he or she do any treatment at the time?

Interviewee: Yeah he pulled a tooth. He pulled one tooth out because it was paining. He couldn't see anything in an x-ray or anything like that. I said well look it's that one in particular. I want it gone. So he pulled it out and had a look at it. He said there's nothing wrong with the tooth. It's just the fact that the gums have receded back and have opened up the porous part of the root.

Facilitator: Yes so you were exposed and that would have been very sensitive.

Interviewee: Yeah.

Facilitator: Did he do any other treatment at the time? Did you have antibiotics or anything else?

Interviewee: They gave me a paste cream to put on the tooth but it didn't work very well. I always turned around and said yeah well I've got to get Sensodyne and go with that. But I never ever did. I just kept holding back. But now I've got to go and get it. I've got to go and get some mouthwash.

Facilitator: One of the recommendations that was given to me - this is sort of a personal experience - is with Sensodyne you just get at night especially at the beginning when you start using it - get a bit of Sensodyne and just put it in the areas that are sore overnight. It helps quite a bit with pain.

Interviewee: Oh yeah okay.

Facilitator: Apart from just brushing regularly with your Sensodyne. Just a suggestion that's all.

Interviewee: Oh great. Sounds good.

Facilitator: Not part of the study but it's my experience.

Interviewee: Well that's how you learn things isn't it?

Facilitator: You talked about your practices. You sort of mentioned your experience going to the dentist. Are you comfortable with the dentist that you have? Is it a private dentist?

Interviewee: It's a private dentist. I don't have any problems with him. He's American. But he's quite good at what he does. The only thing I don't like at a dentist is the cleaning of the teeth.

Facilitator: Yes especially with your sensitive teeth yeah.

Interviewee: Yeah.

Facilitator: You do that through Veterans' Affairs?

Interviewee: Yeah. Everything I do is all done through Veterans' Affairs.

Facilitator: That's good. So you don't have to pay...

Interviewee: I'm covered for war caused injuries or not so it works out well.

Facilitator: Do you face any challenges looking after your oral health or to see a dental professional?

Interviewee: Well I haven't been to the dentist since I've lost the leg. So I don't know what the challenge is going to be to get in there and then get into his chair. I've still got to figure that one out but we'll get it. I'll find a way.

Facilitator: You look after yourself anyway. You brush your teeth yourself.

Interviewee: Yeah.

Facilitator: You don't have any issue with that.

Interviewee: The only time I need help is (1) if I fall in the bedroom. To get back up again I need assistance - or fall anywhere. That's basically it. There was one time last week we couldn't get the chair close enough to the car. I ended up going between the car and the chair and that was very painful. Getting myself back up I had to use the car steering wheel, pulling and almost lost my trousers doing it.

Facilitator: Do you normally just go out by yourself or do you - somebody else with you?

Interviewee: If I'm in the car - I can't drive any more. They took my licence off me. My wife drives. She's with me then. But I do have a four-wheel electric scooter which is quite a little buzz box. I can get around on that very well. That'll take me about 50 kilometres, 60 kilometres.

Facilitator: So like today for your appointment how did you make it to the appointment today?

Interviewee: I get a wheelchair cab.

Facilitator: Oh a taxi?

Interviewee: Covered under Veterans' Affairs.

Facilitator: So if you needed to go to - a dental appointment then you call a cab?

Interviewee: I would have to find my way. Then I would have to do it privately with a cab or - God [unclear] horror if the scooter would make it jump on the scooter and [unclear].

Facilitator: You said it would be done privately so you couldn't get the Veterans' Affairs cab to go to a dentist.

Interviewee: Oh yeah, Veterans' Affairs covers it but it's a private dentist. It's not one at the hospital or anything like that.

Facilitator: Oh right so if it's not a hospital you have to do it privately.

Interviewee: No, no, DVA still covers the private one. They would cover it if it was the hospital. It wouldn't matter. It's just that at the time when I needed a dentist I was over there, he was there and in I went.

Facilitator: I understand. What are your views about including oral health - I think I asked you this before - your views about including oral health during your rehabilitation as part of the education in the program?

Interviewee: Yeah, yeah I don't have a problem with it.

Facilitator: You mentioned that you are okay with a nurse talking about oral health and get assessed?

Interviewee: I can talk to (Rehabilitation Nurse Name Omitted) and (Rehabilitation Nurse Name Omitted) about anything. I feel comfortable. I can tell them my problems. They listen. They speak to me and come up with ideas to try and resolve the problems. Once they're finished and I go out of their office I feel quite comfortable with what's happened. I've got a bit of a spring in my step. Okay I can do that. They're very good at that. I love them dearly for what they do.

Facilitator: Yeah they're beautiful.

Interviewee: I'd be totally lost without them. When I did it before in 2012 all the nurses and physios that were there then - absolutely brilliant. All of the team has been fantastic. I can't fault them.

Facilitator: I'm glad you had a good experience there. The other question is in relation to the information again. When do you think is the best time to get that information? You mentioned leaflet - you did mention television.

Interviewee: I think first off if somebody is going to get it when they first get notified by their cardiologist; you have a heart problem. You need to read this information. If you are interested and are prepared to look after it get in contact with myself so that it can be then followed up and taken further. I think that's where the first instance should lie. The dentist or the dentist's receptionist should start the notification process. Even if for example if you had a website where they could get the information they log on to that website, put their name and all their details in it and get themselves put on to a mailing system where you could mail out the information to them.

Facilitator: A good idea.

Interviewee: That could be started at the dental point and carried on throughout.

Facilitator: Yeah and you mentioned the cardio when you first get diagnosed with a cardiac problem. So through the cardiac doctor and through the dentist as well isn't it - the dental system.

Interviewee: Yeah.

Facilitator: Do you feel you get much information when you go to the dentist in relation to the care you need to...

Interviewee: I don't know about anyone else. When I go to a doctor whether it be dental or body-wise I like to get a good rapport going. I like to treat him not so much that he's a doctor but knowing full well that he is a doctor but hey I'm going to be a friend with this bloke so that I can talk to him, ask questions and get an answer. I think if you work on that basis and you get that rapport going you don't have a problem. But it's up to the individual. Everything can't be handed to you on a platter that's going to resolve all the issues in your life. The person in particular has to put out something to get information and the good stories going back. If they

	don't they're wasting their own time. They're wasting your time. Everybody has more important things to do. That's where I stand.
Facilitator:	Yeah of course I agree with you. You've got to give to get as well.
Interviewee:	Exactly.
Facilitator:	Would you follow the nurse's advice about oral health if they give you advice to see a dentist?
Interviewee:	Yeah.
Facilitator:	Would you follow it?
Interviewee:	Yeah, without a problem.
Facilitator:	Do you feel that nurses are sufficiently trained to provide oral health education?
Interviewee:	One would tend to think if they're giving the information about oral health that they themselves have gone through a lesson or an education program to bring them up to scratch so that they can make these decisions. So yeah I would have complete reliance on them.
Facilitator:	That's the idea. We want to train the nurses. This is part of what we're trying to do there. Do you feel the nurses are sufficiently trained now to give you specific information about oral health for example?
Interviewee:	In relation to (Rehabilitation Nurse Name Omitted) and (Rehabilitation Nurse Name Omitted)?
Facilitator:	All the [unclear] you have.
Interviewee:	Yeah, I would say yes. They could gather the information. They could pass it on. I wouldn't have a problem at all with them looking in my mouth and turning around and saying; I think that's a problem in there. You need to go and get it checked. That's cool
Facilitator:	I was suggesting - and that's very good - I was considering that you haven't received information so far so it is to hopefully create this thing that nurses will be in the head of everybody and incorporated into either post-surgery, before surgery or if people don't have surgery also at some point get that information that's important.
Interviewee:	Yeah.

Facilitator: We have already spoken about your social support. I wanted to talk about who is supporting you socially.

Interviewee: Yeah I've got the most beautiful wonderful wife going. What she does for me is amazing.

Facilitator: The other question is if there was a free dental service that ran regularly, offered to people with cardiac conditions would you attend it?

Interviewee: Yeah.

Facilitator: Have you got any comments or suggestions that you may say about our conversation today? This is the last question.

Interviewee: I've got nothing to say in retrospect. But everything that I would have said I think I've said during the interview.

Facilitator: Yeah you've said a lot. Thank you.

Interviewee: It's something that - hey if there was an endorsement needed I would endorse it. I've gone through quite a bit in my life. To find some group, panel, body, whatever, that's coming out with this good information - it's information that is well worthwhile. As we said earlier the link between oral health and cardiac was never ever known. Now it's coming out. I think it's something that's got to be put out there and shouted. This is possibly happening to you; and brought into that field. I think if that can get through and it can go all the way, what a way it's going to be.

Facilitator: That's beautiful.

Interviewee: It's going to bring in a knowledge bank to everyone that's available.

Facilitator: Yeah, and in prevention - preventing - people dying basically because some big studies they've done in the United States - one of the studies that - people that have recurring infarcts when they have poor oral health. Thank you very much (Interviewee Name Omitted). Thank you.

Interviewee: You're welcome (Facilitator Name Omitted). Thank you darling; much appreciated.

Facilitator: It has been a pleasure talking to you and it's been a pleasure meeting you as well.

Interviewee: Yeah.

[Aside discussion]

Interview 5

FILE DETAILS

Audio Length: *14 minutes*

Audio Quality: ☒ *High* ☐ *Average* ☐ *Low*

Number of Facilitators: *One*

Number of Interviewees: *One*

Difficult Interviewee Accents: ☐ *Yes* ☒ *No*

Other Comments:

Facilitator: Thank for agreeing to do the interview and just to confirm that you're happy for us to record the interview.

Interviewee: Yep.

Facilitator: The first question that I'd like to ask you is about your diagnosis. How long ago were you diagnosed with your cardiac condition?

Interviewee: Six months ago.

Facilitator: Six months ago?

Interviewee: Yep. I had the operation three months ago.

Facilitator: So you had a bypass?

Interviewee: No I had an ascending aorta and they changed the aorta.

Facilitator: Okay. Is this the first program that you've attended, the cardiac rehabilitation program?

Interviewee: Yep.

Facilitator: First time. My next question is in relation to the study. Have you ever heard anything about oral health and cardiac disease or cardiovascular disease?

Interviewee: I was made aware of that when I was told that I had to go for an operation. I was made aware that I should go see a dentist. They had to give me a clearance in case there were infections or something there. So I was made aware of that.

Facilitator: Did he give you any more information apart from that?

Interviewee: Not really, no.

Facilitator: Did they give you any written information about…

Interviewee: No.

Facilitator: So it was just verbal was it?

Interviewee: Yes.

Facilitator: Who was the person who told you about it?

Interviewee: What do you mean, told me about…

Facilitator: You said you heard it the first time, was it a doctor or a nurse?

Interviewee: The surgeon's secretary rang me and said the doctor would like you to go see a dentist to make sure there's no infections in the gums and that.

Facilitator: Before the surgery.

Interviewee: Yeah about a week before, yeah.

Facilitator: Okay. Do you think that poor oral health can affect the heart?

Interviewee: I didn't think it could, but apparently it can.

Facilitator: Do you know how it affects it?

Interviewee: No.

Facilitator: That's okay and that's a really common answer. Most people don't know. Obviously you now know that there may be a relationship between oral health and cardiovascular disease.

Interviewee: Yeah.

Facilitator: You mentioned you did get information verbally. So apart from that have you received any other oral health information from any cardiac group either in hospital, outside hospital, rehabilitation? None.

Interviewee: No.

Facilitator: If there was information would you like to get some?

Interviewee: Yeah it's interesting to know. Yeah.

Facilitator: For information that was given to you, what do you think is the best way of receiving it? Some examples would be leaflet, pamphlets, written, verbal, internet. What would be the best?

Interviewee: I'd probably prefer a leaflet or pamphlet I think. Yeah.

Facilitator: Was the best way of receiving that information?

Interviewee: Yes.

Facilitator: So can you tell me about your oral health. Having you got any current concerns about your oral health?

Interviewee: No not at all.

Facilitator: None at all. Okay.

Interviewee: No.

Facilitator: Do you see a dentist regularly?

Interviewee: Well before my operation I had to go to get them checked and that. That was my first time that I went to a dentist.

Facilitator: You haven't gone after that?

Interviewee: No.

Facilitator: So this is about six months ago?

Interviewee: Three months.

Facilitator: Three months ago sorry yes, before the surgery you said. Yeah.

Interviewee: Yeah. I'd never been before that.

Facilitator: What are your normal oral habits? What do you normally do to look after your teeth and your mouth?

Interviewee: Just brush my teeth.

Facilitator: How often?

Interviewee: I'm not going to say every day because I'd be lying but I usually when I'm in the shower and I'm at home. Maybe oh, look at least every second day.

Facilitator: Yep and brush your teeth, anything else?

Interviewee: No, just brush. Yep.

Facilitator: No problem. So you told me three months was the last time you saw a dentist?

Interviewee: Yep.

Facilitator: How was your experience when you went to the dentist? Have you got any fears or face any challenges?

Interviewee: No it's fine. All he did was he gave them a clean and that was it. That was all good.

Facilitator: Do you see any issues seeing a dentist; do you see any challenges for you?

Interviewee: No not really.

Facilitator: No? So if you wanted to see somebody, you just go, you don't worry about it?

Interviewee: Until I get a toothache, no I don't worry about it.

Facilitator: So you'll see one when you have a problem. Is that what you're saying?

Interviewee: Yeah I think so.

Facilitator: Okay, that's cool.

Interviewee: Well I didn't see one for 49 years so…

Facilitator: You've never had any problems?

Interviewee: No.

Facilitator: What are your views about including oral health information why you're attending cardiac rehabilitation or cardiac program?

Interviewee: I think it's such an important part of cardiac, I think it should be included there if you're telling me that it's an important part.

Facilitator: It is. What about a nurse giving you information? How do you feel about a nurse assessing you, asking you questions about your oral health and also assessing your mouth just having a visual? How do you feel about that?

Interviewee: Yeah. No problems with that.

Facilitator: You're comfortable?

Interviewee: Yeah.

Facilitator: If a nurse will refer you to a dentist would you follow that advice?

Interviewee: If she says that I need to see the dentist - yeah.

Facilitator: So you're basically comfortable receiving information from a cardiac rehabilitation nurse or a cardiac nurse in relation to oral health and you're happy for that person to do an assessment on you and refer you

if necessary? When do you think is the best time to receive that information?

Interviewee: What do you mean?

Facilitator: Since you were diagnosed with your cardiac condition. When do you think would be the best time to receive information about the oral health and cardiac problems or relationship? When is the best time to get that information do you think? Before the surgery, after the surgery, during rehab?

Interviewee: Well usually before the surgery, from what I know what they did with me, they make sure that they're checked and okay. So after that I'd say after the surgery, maybe six months or something.

Facilitator: Yes because you mentioned they told you to go and see the dentist but you didn't get any information specifically about what's the relationship.

Interviewee: No all they said - when the specialist's secretary rang and she said for this operation we need to check your gums and teeth. So you need to go to a dentist to get a clearance letter. That's when I was made aware that teeth had something to do with the cardiology. Otherwise I wasn't aware of that at all. But what you're saying is after, for regular checks after, it's a big operation and a long recovery period so that's probably the last thing on your mind. So probably six months later or something I'd say.

Facilitator: Yeah. I was asking you about getting information or getting a leaflet or getting more details of what is the relationship and what…

Interviewee: Everybody's different. Me personally, I'm the type of person, when I get something in the mail or something, I always read it no matter what it is but some people might just chuck it away. Yeah.

Facilitator: That's true. Do you think nurses are sufficiently trained to teach you about oral health?

Interviewee: Oh yeah of course I think.

Facilitator: Yep? So you're happy with the nurse…

Interviewee: They should know shouldn't they if they've done their study as nurses, they should know that.

Facilitator: Yeah actually to be honest the nursing course doesn't actually include oral health. So nurses do know…

Interviewee: But they should if they're going to work in the cardiac ward they should know then, shouldn't they? They should've studied that.

Facilitator: Yes they should and that's why we're doing this project. Unfortunately this is not happening. That's what we want to be included. That's part of the aim of project, to include the training for the nurses. So yeah you're right, they should. Can you tell me about your social network and your support, are you married, family?

Interviewee: Yeah married with kids. Dogs, cats, the whole works.

Facilitator: You look very independent. You've come by yourself. Do you drive?

Interviewee: Yeah I'm fine now. Yeah.

Facilitator: Do you drive or come by public transport?

Interviewee: No I drive.

Facilitator: Even when you had the surgery, your main support was?

Interviewee: My wife.

Facilitator: Okay good. Did it take you long to recover from…

Interviewee: I was in hospital for about 11 days and then at home, before I become independent, was probably a good two weeks. Yeah so three, three and a half weeks all up.

Facilitator: It wasn't that long.

Interviewee: Yeah.

Facilitator: If there was a free dental service offered regularly for cardiac patients would you attend it?

Interviewee: When you say one run regularly?

Facilitator: Like for example if there's a system where they say every six months they are going to do a check and clean out every year.

Interviewee: Yeah well I decided after I was made aware of this, I decided now you know I'm nearly 50 years old, I'd never been to a dentist before. So now I've decided that once a year I'll go and get a check-up and a clean or something.

Facilitator: When you go to do this do you own appointment privately or do you go through the public system?

Interviewee: Well I did last time private, yeah.

Facilitator: Do you find that a problem? Organising the appointment and paying for it?

Interviewee: Not really.

Facilitator: No? You don't find it expensive or - it's okay?

Interviewee: I can't remember how much it was but…

Facilitator: Yeah that's fine, that's good that. So do you have any problems…

Interviewee: I know a lot of people don't go to the dentist because they are expensive. I know a lot of people don't go.

Facilitator: It's true. It's another issue with people, the waiting. People sometimes have problems with waiting to see a public dental service.

Interviewee: Yeah.

Facilitator: Have you got any other comments that you would like to make in relation to what we have been talking about and our plan?

Interviewee: No that's fine. I think if the teeth and gums play a very big part in that there should be more awareness of that. Like I said, me personally, I'd never heard of it before. I was told before the operation yes, so I'm sure there's a lot of people like myself out there then that wouldn't…

Facilitator: Understood. It's true, unfortunately that's why we started this.

Interviewee: I can only speak on my own behalf. I had no toothaches, my teeth were fine. I've still got all my teeth so my attitude was why I should go to a dentist. But if I knew that it could affect something else then I probably would've gone every two or five years or something.

Facilitator: Yeah of course. Okay, thank you. I think that's it. That's all we wanted to talk about. Basically why we started this project is there's no information for patients on this strong relationship between poor oral health and cardiac disease. Especially people that already have problems.

Interviewee: Like I said, I was amazed with that. When I got this phone call that said you've got to go to the dentist. I'm on the phone going what? Dentist?

What for? You know I've got a sore heart not sore teeth. I was shocked. Yeah, I didn't know.

Facilitator: There you go. Thank you very much Michael.

Interviewee: No problems.

Facilitator: I hope you make it to your next - you have an appointment at 11:30 you mentioned.

Interviewee: Plenty of time yeah, it's only 10:30.

Facilitator: Okay thank you very much.

Interviewee: Thank you very much. All the best.

Facilitator: Nice to meet you. All the best.

Interviewee: Merry Christmas.

Interview 6

<u>FILE DETAILS</u>

Audio Length: *20 minutes*

Audio Quality: ☐ *High* ☒ *Average* ☐ *Low*

Number of Facilitators: *One*

Number of Interviewees: *One*

Difficult Interviewee Accents: ☐ *Yes* ☒ *No*

Other Comments:

[Aside comment]

Facilitator: Okay, so thank you again for agreeing to participate with this interview. I guess just to confirm that you're happy to continue on with recording.

Interviewee: Yes.

Facilitator: Yeah, you are happy?

Interviewee: Yes, most definitely.

Facilitator: Okay, thank you. So just to start, can you tell me a little bit about your diagnosis and how long ago was that?

Interviewee: I've been diagnosed with end stage dilated cardiomyopathy. It's been about two to three years - 2012, so about three years.

Facilitator: Three years now.

Interviewee: Yeah, three years.

Facilitator: Are you on a waiting list for a transplant?

Interviewee: No.

Facilitator: No, okay. Is this the first time you've attended the cardiac rehabilitation program or cardiac program?

Female: Yeah, we've been here a while now.

Interviewee: We've been here a while now. About seven months.

Facilitator: Seven months so you had…

Interviewee: It's pretty much ongoing yeah, well pretty much because of the diagnosis, it's just ongoing.

Facilitator: You're happy with everything here?

Interviewee: Very happy.

Facilitator: Comfortable?

Interviewee: Yeah, very comfortable.

Facilitator: Oh that's good. You're from this area aren't you?

Interviewee: Yes, which is probably why it enables us to come every two weeks or sorry, twice a week.

Facilitator: Twice a week. Yeah, I noticed you come quite often. Okay, thank you for that. What do you know about oral health in cardiovascular disease? Have you ever heard anything about it since you've been diagnosed?

Interviewee: I haven't heard anything. As far as my knowledge goes in regards to oral health and heart failure, I think it's more the germs, we're more prone to getting sick. Obviously the teeth open up to a lot of germs and stuff, so I can only imagine that's maybe something about it. As far as checking my teeth or getting teeth whitening or what I don't know.

[Laughter]

Interviewee: But no, that's as far as I know.

Facilitator: You say that's all you know, have you ever heard from anybody about it or just what you heard?

Interviewee: No, no. Just looking up. When you're dying you want to look up anything and everything that can help you prolong your life. I know for a fact that germs - I have to try and keep myself as clean as I can, disinfect my hands, brush my teeth as best as I can, try and keep away from as much germs as I can. That's what I mean, so that's what I've looked up in regards to heart failure, it's just about trying to be as healthy as you can. Keep your heart pumping, that's why you see me training in there. I train hard because life's too short. Obviously. So it's just do what you can do. If you've got a toothbrush you use it.

[Laughter]

Facilitator: That's right. So nothing written, nothing [unclear]…

Interviewee: No information packs or anything, just Google.

Facilitator: So obviously maintaining your oral health is important to you?

Interviewee: Very important. You can only try and do as much as you can. With this disease it's hard too, because sometimes you wake up and you're not able to get up out of bed for a few days, so it is hard. But when you are able to do it, I do it.

Facilitator: Yeah, does that happen often that you feel…

Interviewee: It's probably on average two to three days per week if I'm feeling like I just can't do anything. But I base my whole fitness and basically all my energy levels are based on the race, as you can see. Next week I've got another two appointments, next week after that I've got another two, so my whole activities is based on those appointments. Like today the race, I go home and rest. Then tomorrow I wake up and go for my walk as well. So it's all just basically our own program which is pretty much our full-time job now. It's just monitoring our health and our foods, our water intake, everything.

Facilitator: I can see you're very lucky your wife is with you.

Female: Depends what day you ask him.

Interviewee: Oh very, very.

[Laughter]

[Over speaking]

Interviewee: No, no, very lucky.

Facilitator: So you said no information. Because you haven't received any information, the next question is would you like to get more information about the relationship between oral health and cardiac disease?

Interviewee: Oh most definitely. Most definitely.

Facilitator: If you do receive information how would you like it delivered?

Interviewee: Email, paper, documentation like booklets, little booklets or something. I'm always interested, especially being diagnosed with what I have, I'm always interested because it doesn't matter, if your mouth is sick basically your teeth are hurting, it all reflects the heart. So the less pressure we can apply on the heart the easier it will be for me, so that I do know. I know sometimes my teeth hurt. There is nothing I can do about it. I'm not able to take certain medications because of my heart medications that I'm on.

Facilitator: Have you got any current concerns about your oral health or your mouth or your teeth right now?

Interviewee: No.

Facilitator: No, you're okay with everything?

Interviewee: I'm just missing teeth but that’s just normal.

Female: Not missing [unclear].

[Laughter]

Interviewee: But yeah, no real concerns.

Facilitator: When was the last time you saw a dentist?

Interviewee: Yeah, good question. Probably would have been about six years or seven years ago just [unclear] reasons. Just had one of my teeth ripped out, that's the only reason I saw the dentist.

In regards to the regular check-ups, even when I wasn't sick, I was working. It just wasn't feasible for me to go and do check-ups at dentists. Even now we just spent a lot of money on my kids' braces, that's more important to me than my own teeth if that makes any sense. I would rather spend $1000 on my kids' braces than $20 on me for a

check-up. It's just the way I am. So yeah, but hey I love free things. If they're going to be offering it for free and if they're going to check, I'll check my teeth.

[Laughter]

Facilitator: I mean you are aware that there's a service through the health system, the public health system that you could do. Have you tried that?

Female: No I didn't…

Interviewee: No, we didn't know. We were just told that the dentist was just no go for any public or health system.

Facilitator: You were told?

Interviewee: We were told that.

Female: Yeah, that was a month ago though.

Interviewee: We were just - we can't afford - a lot of people can't afford $250 for a check-up. I mean that's 50 per cent of normal people's rent per week. So if you think about it, do I want to worry about my teeth or do I want to pay my rent, I think I want to pay my rent. So yeah we were - I don't know if we were told but it's just human nature - it's just we think that we were told.

Female: Every time you go to the dentist, you're out of pocket [$100].

Interviewee: Dentist is no, you've got to pay for that. We just never went.

Facilitator: I'm not sure, perhaps you can follow that up and see because having the condition that you have you may be able to actually have free access to [unclear] cleaning at the hospital, there's one in (Suburb Name Omitted).

Interviewee: We just never have come across it, yeah we'll look into it. Like I said, I've got heart failure, I don't think rotten teeth or a sore tooth is going to be anything of a hassle for me. But like I said, it's just mainly my kids and my family, my wife that's more concerning to me about their teeth and stuff. Because when you're in a coffin nobody looks at your teeth when you're dead.

Female: It depends if [unclear].

Facilitator: I guess it depends how soon you want to get there.

Interviewee: Yeah.

Facilitator: So I guess if you look after…

Interviewee: Just as long as I get my hair cut, I'm happy.

Facilitator: You look good [laughs]. So how often do you - what type of dental, what do you do for your oral health regularly?

Interviewee: I just brush my teeth twice a day in the morning and night. When I can remember I floss.

Facilitator: You floss as well?

Interviewee: Yeah. That's it. At times, it's so funny because I used to get so tired. Because of this illness, I get tired brushing my teeth. I get breathless brushing my teeth. So my wife went and bought me an electric toothbrush.

Facilitator: Oh right.

Interviewee: An electric toothbrush and yeah it was…

[Over speaking]

Interviewee: Oh yeah it just makes it because of the motion that you're brushing your teeth - it's just tiring. It's hard to believe that you can get tired brushing your teeth but with heart failure it's really - sometimes I feel like you see me up in there and then I could drink a coffee, I like coffee, or my tea at night and then just get so exhausted by just…

Facilitator: Really feel it?

Interviewee: It's just exhausting. Or just standing up. It's just weird because some days I feel like I could walk for an hour and a half or two hours.

Facilitator: You have to enjoy it, all the good times?

Interviewee: Everything. Exactly, that's what I do. Yep.

Facilitator: So you mentioned that when you went to the dentist last time, you had an extraction, you had a tooth out. How [did it feel], can you tell me a bit more about it? Did you have any problems going to the dentist?

Interviewee: Definitely no problems going to the dentist. The experience was good. I had to get it out. It was numb, but it was good because it was just my excuse to get people not to talk to me. I like to be alone most of the time.

Female: What did you say when you came home, I think it [unclear]…

Interviewee: It was just so sore like when it wore off.

Facilitator: It would be yeah of course.

Interviewee: But [unclear] I think.

Facilitator: You've mentioned some of the challenges seeing a dentist. You mentioned about money. Any other challenges seeing a dentist?

Interviewee: No, not really. As far as challenges go, I think it's just more the obstacle in regards to the finance. Like I said, I don't see many people wanting to go and see a dentist and pay $250. I just don't see it logical for someone and then their rent is $500 a week. It doesn't make any sense when you can just leave your teeth, let it rot and let it fall out. You know what I mean?

As a father I wouldn't - maybe as a wife because my wife has had that and I've sent her to the dentist straight away because I don't want my wife to experience that pain. I've found someone beautiful why do I want to keep my teeth [unclear] you know what I mean. So it doesn't faze me anymore. I've got three kids that I've got to worry about, they're my priority. But those are my challenges stopping me from going to the dentist.

Facilitator: So what are your views about including oral health in the cardiac setting or in rehabilitation?

Interviewee: I think it's definitely great in regards to - I think any exposure of the heart patients or the patients in general that they have in hospital, it gives them not only a - it will give them a boost because number one, it will give them something to do, number two they are close by someone that's medically trained, which will help especially if they know how you're feeling. Just being able to have contact with someone that is medically trained and certified that you [often regard] whether it's for your teeth or for your heart, it will boost them.

When you're looking good you feel good, and if you feel good it's just a domino effect because that's only a positive thing. I can only see it's going to be a positive. I just don't see any negative thing.

Facilitator: How do you feel with the nurses giving you that information? Do you feel comfortable with nurses telling you about oral health?

Interviewee: Yeah because it helps. Any information that you're getting, it helps someone else. Maybe it won't help me, but it will help someone else that doesn't know. Someone that may have missed this class or missed the information that you've come around with today, last week. They turn up next week and they may have missed it but they may be interested, so therefore I'm sort of helping them. It's just a matter of helping people. The Government is getting a 2 per cent pay rise next year, the Prime Minister is getting a $10,000 pay rise at the end of the year and we can't even get a $250 free health check for our teeth. You work that out. So anything that's there that we're entitled to that us tax payers work for I reckon we should bloody well damn get it. Full stop.

Facilitator: So do you feel comfortable with the nurses - you said you feel comfortable with the nurses asking you questions.

Interviewee: Yep, most definitely.

Facilitator: What about [doing a visual]?

Interviewee: Oh that's fine, look I mean I'm…

Facilitator: If they want to have a look at…

Female: They've seen worse.

Interviewee: The nurses seen me butt naked when I was in intensive care in the heart failure ward so it doesn't bother me.

Facilitator: So if a nurse refers you to a dentist to give you follow-up advice?

Interviewee: That's absolutely fine, yep. Anything to support, we're there. Anything to support the program and it's something that's definitely needed. My personal opinion, I think that's definitely something needed. It would give them a good feeling. Something like painting the room would just give you a good feeling. When you feel good you get up. If you wake up to messy house you're just going to have the worst day. If you wake up to a nice clean house you're going to have a beautiful day.

Facilitator: That's good. So if there was a free dental service offered to cardiac patients as a regular service, would you attend it?

Interviewee: I would definitely attend it. That's part of my full-time job now is my health and your teeth is part of your health which is connected to your heart, so of course I would be.

Facilitator: Just making sure I'm not missing any questions. You said before the nurses are alright. Did you feel that the nurses are sufficiently trained to give you advice and tell you about oral health?

Interviewee: I think those nurses that are trained and qualified are sufficiently experienced enough to give me advice. I don't know who the nurses are. Are you talking about in regards to the [unclear]?

Facilitator: Cardiac nurses.

Interviewee: Cardiac nurses. Oh most definitely. Most definitely because I can only imagine that in order to become - I'm no nurse or anything, but I know that the heart is your body function, it's the main muscle that functions your whole body. Teeth is part of your body, so if you're not going to get trained in your cardiac medical professional that something has got to happen with your teeth, I think there's something missing there. So most definitely I think they would be. Maybe just a refresher course. I think they'd be most definitely. I will take advice from someone that's a cardiac nurse that's giving me advice in regards to my teeth than a dentist. A dentist doesn't know. A dentist is only specialised in teeth. Cardiac nurses specialise in not only the teeth but the functioning of the heart.

They know you can do that but you don't do this, take a little bit of this and that. The dentist is like until you tell him that you've got heart failure then he has to doubt all his - he will have to adapt the way he's doing it because he can't do the same thing he would do with a normal patient than on a patient with heart failure. So no, I feel more comfortable coming to the nurses most definitely.

Female: The nurses are familiar as well, because you're in and out of the hospital with programs and stuff here. You're quite familiar with some of the nurses, so all ears for whatever they have to say.

Interviewee: Yeah and I think nurses are a lot better looking than dentists.

Female: Yeah our dentist is pretty good-looking.

Facilitator: Better looking. Male and female nurses, right?

Interviewee: Oh both, yeah.

[Laughter]

Facilitator: So tell me a little bit about your support. I can see your wife is your social support and any other support that you have?

Interviewee: No, my only support I have is my wife. I have family members but we won't get into that. My support is just my wife. My wife has been there through the good and the bad. We've been together, we're married obviously. So I mean that's full stop. My kids are supportive sometimes when they are good but maybe they are keeping me healthy because every time they do something bad at school I get angry and when I get angry I start to feel more strong and fit.

[Laughter]

Interviewee: But no, as I said my only support is my wife and that's all good, and of course the nurses and the cardiologists and (Name Omitted) Hospital have really been our support because they have been wonderful. They saved my life. There's a story to it, but the nurses in there saved my life. As long as I'm still here I'm going to not let it go to waste. I'll be supporting every program that (Name Omitted) Hospital has to offer because it can only benefit - you never know my own kids could end up to where I am and what we're about to do would benefit them and the our kids, our nephews, our nieces. We don't know.

Facilitator: That's right. If future generations…

Interviewee: Life circles around - what we do today probably won't benefit us but it could probably benefit your sister, your brother, your mum, your dad. You don't know what's going to happen.

Facilitator: When you do come, do you come by public transport or drive?

Interviewee: My wife drives.

Facilitator: You drive. You're not driving?

Interviewee: No, I'm not allowed to drive. Yeah, but my wife's my full-time carer now.

Facilitator: Your children - how old are your kids?

Interviewee: Nineteen, 16 and 13. I think.

Female: Yeah that's it. You got it.

Facilitator: Oh good ages.

Interviewee: Yeah, good memory.

[Laughter]

Facilitator: Is there any other comments that you want to make in relation to what we've been talking about and the possibility of having a program?

Interviewee: No. First and foremost I just want to thank you for going out of your way and taking time to do what you do. I don't know what started this off but it's most definitely a great thing. Like I said, heart failure - it's definitely a good stepping stone to someone with heart failure, because when you're diagnosed the first thing you're thinking is that you're going to die, when are you going to die or how are you going to die. The little build up things, like I said little nurses rubbing your hands and just making sure you're okay, that goes a long way.

Dental care making me feel good, that goes a long way. Like they do in nursing homes when they have make-up artists come and make up people, when they come and do the make-up for the elderly people. It gives them a good feeling. They get their haircuts. It gives them a good feeling. They might be dying next week but at least they're happy. Just that good feeling and the memories that come back of when you were young and stuff like that. I think it's a great thing and so I want to thank you…

Facilitator: No, thank you.

Interviewee: … for allowing us to take your time.

Facilitator: No, thank you for being here. That's the idea, to hopefully to make it better for patients.

Interviewee: Definitely.

Facilitator: There is a strong relationship and there is a problem with people that have poor oral health can affect the heart, especially if they already have heart problems. So infarcts, strokes and all those things can happen recurrently. That's an area to incorporate into the cardiac system and to become a normal referral pathway. So that's the idea, on Medicare so people don't have to pay, because I think that's the issue for many people. So thank you very much.

Interviewee: No great. That's great. Thank you.

Facilitator: I wish you all the best.

Female: Thank you very much. Good luck.

Facilitator: I might just turn this off.

Interviewee: You have a good Christmas.

Facilitator: You too. I wish you the best and I'll see you around. I'll be coming again.

Interview 7

FILE DETAILS

Audio Length: *25 minutes*

Audio Quality: ☒ *High* ☐ *Average* ☐ *Low*

Number of Facilitators: *One*

Number of Interviewees: *One*

Difficult Interviewee Accents: ☐ *Yes* ☒ *No*

Other Comments:

Facilitator: Thank you for participating in the interview, and just to confirm that you are happy to continue with recording?

Interviewee: Yes I am.

Facilitator: Thank you. (Name Omitted), you mentioned that you had a bypass done?

Interviewee: Yeah five bypasses.

Facilitator: Five bypasses. How long ago was that?

Interviewee: Basically the first week of June.

Facilitator: This year?

Interviewee: Yeah.

Facilitator: Have you had any heart problems before that or been diagnosed with any cardiac condition?

Interviewee: No, short of breath, which I put down to other things, and never had checked, until February when I had an argument with the boss's wife. I

had hot and cold sweats, couldn't stand up out of the chair, banging in the back of the head. I realised that something was terribly wrong. So I basically went home, they put me into (Name Omitted) Hospital and they did all the tests. But because I didn't have any pain in the arm, the chest, back, shoulder, they said I was that close to a heart attack and probably that close to a stroke, but I didn't have either.

So then they gave me angiogram. Hopefully that was going to be a stent and go home happy, but they went in and had a look and said, can't do anything for him, too blocked up. So then I went for bypass surgery. That was done actually on 25 May.

Facilitator: 20 May, 2015?

Interviewee: 2015.

Facilitator: Big story.

Interviewee: So pretty quick, yeah.

Facilitator: It was so quick. You're now attending - you're just finishing cardio rehabilitation this program. Is this the first program you attend?

Interviewee: Yes.

Facilitator: The six weeks program isn't it?

Interviewee: I've probably been coming for longer than that because quite often I miss the Monday. I get up in the morning and I just can't get up. So I end up sleeping and end up missing it. But yeah I just - and they said I could just keep coming until I feel okay.

Facilitator: So when did you start coming, do you remember?

Interviewee: Six weeks to the first check up, I didn't do anything then, and then it was another month. Then we went to America, which we'd all bought and paid for, we couldn't get out of it so we went to America, were there for a month. Came back, so probably August, September, it would have been probably mid-September when I started coming.

Facilitator: You're happy with the program?

Interviewee: Yes.

Facilitator: Good.

Interviewee: Otherwise I'd just sit at home watching TV.

Facilitator: So it's good that you have something to do?

Interviewee: Yeah.

Facilitator: So talking about the reason for the interview and the study that I'm doing, what have you heard or have you heard anything about oral health and cardiovascular disease?

Interviewee: Nothing. I'm a diabetic as well and one of the doctor's said that diabetes and oral health, there was a link between them. But I don't know anything else.

Facilitator: Nothing about heart?

Interviewee: No.

Facilitator: Not before and not after your…

Interviewee: No.

Facilitator: So there's no information and you don't have any idea of any relationship?

Interviewee: No.

Facilitator: Do you think that poor oral health can affect the heart?

Interviewee: I wouldn't think so.

Facilitator: Is maintaining oral health important to you?

Interviewee: I could say yes, but I don't visit the dentist, I hate the dentist.

Facilitator: [Laughs] you hate the dentist? When was the last time you went to the dentist?

Interviewee: Actually between having my turn and having the operation, sometime between February and May, I did go because one of the teeth at the back was giving me trouble. Again all they wanted to do was pull the bloody tooth out, which I don't have enough teeth left to afford to pull them out and I insisted he - and I was on blood thinners, so I didn't really want him to pull a tooth out while I was on blood thinners. So miraculously he gave me antibiotics and he plugged it up, filled it and I haven't had any trouble since. So I'm a bit annoyed that the first thing they want to do is pull your teeth out, without doing something with whatever's there.

Facilitator:	So you went by yourself, nobody told you to go you just went because you had the [unclear].
Interviewee:	I had the ache yeah, I had to go, course the wife's on me back about going.
Facilitator:	[Laughs] It wasn't because you were told by any health professional or nurse or doctor?
Interviewee:	No.
Facilitator:	You just went, and you haven't gone back since then?
Interviewee:	No.
Facilitator:	How do you look after your oral health?
Interviewee:	Basically after breakfast I toothbrush and before I go to bed, toothbrush.
Facilitator:	Brushing teeth, that's all?
Interviewee:	Yeah I've now got one of those electric toothbrushes, that's got the two bit heads, it seems to work okay.
Facilitator:	So maintaining your oral health is important to you obviously?
Interviewee:	Yeah but having said that I'll walk up to my wife and she'll say oh get away from me your breath stinks. But I can't smell it so I can't smell that my breath smells. I don't know if you get used to it or something. Obviously I must have something else that needs looking at.
Facilitator:	Maybe.
Interviewee:	But I don't want to go.
Facilitator:	But usually we have a bit of bad breath in the morning don't we? [Laughs]
Interviewee:	Well I suppose you can, it depends what you eat too I suppose.
Facilitator:	So have you ever received any oral health information in the past?
Interviewee:	Not really no.
Facilitator:	Not specifically. Would you like to receive any information?
Interviewee:	Oh yeah, something on your health is always helpful.
Facilitator:	If you do receive information how would you like it delivered? How would you like the information to be given to you?

Interviewee: Oh probably by mail but my wife does have an email address. We could get it by email yeah.

Facilitator: Email. Have you got any current concerns about your oral health, your mouth, teeth?

Interviewee: I think there's something wrong because every now and then I get a little twinge on one of those teeth, so I've got to go and get it looked at. I'm thinking about that.

Facilitator: Thinking about that [laughs] okay. You mentioned when was the last time you went to the dentist, your health practices; you did tell me a little bit about your experience with the dentist. You did mention that you hate going to the dentist.

Interviewee: Yeah originally when I first was married back in the '70s, there was a dentist in (Place Name Omitted) who I went to and immediately pulled one of my teeth out. Within about three months, the tooth next to it collapsed. So he pulled that bloody tooth out. Then I find out talking to a few people at a barbecue, don't go to him all he does is pull your teeth out. So a guy said I go to a guy down in (Place Name Omitted). I went to him, (Name Omitted) his name was an older fellow, he said oh no I never pull teeth out. We always try to repair the tooth even if we've got to give you - and then I had some of that, what do they call it, root canal therapy, where they take the root out and then fill the tooth up. Yeah, he never ever pulled my teeth out, but he's retired and gone. Since then I've had teeth collapse to the point where you can't really save them.

Facilitator: You said you've got diabetes how long have you had diabetes for?

Interviewee: I've probably had it for it 10 years but I only got diagnosed about three years ago.

Facilitator: You're on medication, tablets or injections?

Interviewee: Tablets.

Facilitator: Just tablets and diet yes?

Interviewee: Yeah.

Facilitator: Because I'm curious about that. Do you face or do you see any challenges visiting a dentist? What would be some of the issues that you face seeing a dentist, or barriers that…

Interviewee: I think basically I don't want my teeth pulled out and I don't want the pain. Although the last time I went for this last little bit of work were he filled it, there wasn't any pain. The needles are so thin now you can hardly feel them go in, it's all numb. I suppose they are a lot better nowadays. That was the local guy there at, I suppose you'd call it (Place Name Omitted), prior to that I'd been to dentists at the (Dental Clinic Name Omitted) here in (Place Name Omitted).

Facilitator: (Place Name Omitted), yeah the 24 hour emergency.

Interviewee: Yeah but they've also got the really high 24 hour paying scheme as well. They seem to charge a lot more. So I went to the little guy there at (Place Name Omitted) and he seemed to be more reasonable with his charges, plus it was all new equipment. Not as nice as the equipment in (Dental Clinic Name Omitted) where you could actually look at the screen and see your whole - when he took the x-ray, it was instantaneous. It was on the screen you could actually see it all in front of you but he was on par and he had some reasonable staff there yeah.

Facilitator: So cost is important to you isn't it?

Interviewee: Well yeah, I've never had money so I've always had to make my money go as far as possible. I tend to probably shop around and get the cheapest one.

Facilitator: Have you ever gone to a dentist or tried to organise dentist through the public health system?

Interviewee: No.

Facilitator: But you're aware that is available?

Interviewee: Well, I probably am but I've never ever read about it or looked at it. I am aware that we're with (Health Insurance Name Omitted), before I came down with diabetes, my wife who's now working for (Place Name Omitted) and starting to earn some good money - because I'm not working at all now thank god. She did the right thing and went and joined us up into (Health Insurance Name Omitted) which worked out pretty good with this heart operation because I paid nothing basically.

Facilitator: Oh that's good.

Interviewee: A couple of thousand maybe. But all the rest of it was paid for by (Health Insurance Name Omitted). That would have really cut into my money if I'd had to pay for all that as well.

Facilitator: They cover for dental as well?

Interviewee: They do have - yes that's where I was going with that. (Health Insurance Name Omitted), have a dedicated dentist and I think it's at (Place Name Omitted). (Place Name Omitted) is a pain to get to. I've got a car I could drive there but the parking and all the rest it's a pain. So I haven't been to them, I'd just rather go to the local one you can drive up park in his front yard and go in and get it all done and dusted.

Facilitator: What are your views about including oral health education and talking about oral health during your rehabilitation or in the cardiac setting?

Interviewee: Sorry?

Facilitator: What are your views, what do you think about getting information about oral health...

Interviewee: Oh right yeah.

Facilitator: ... when you're attending your cardiac rehabilitation program?

Interviewee: Well I didn't think there was any correlation between the two, but obviously there must be. So yeah look, I'm one of those people that believe if you don't learn something new every day you've wasted a day of your life. So if there's something new to learn, then I'm quite happy to receive the information.

Facilitator: So how do you feel about the nurse giving you that information?

Interviewee: Oh yeah definitely.

Facilitator: Are you comfortable?

Interviewee: At (Name Omitted) Hospital the nurses there were absolutely excellent. They would bend over backwards to help you, right down to when I was about to leave and the girl was writing up my chart. She came in and ran through everything, item by item by item. Touched on different subjects that I should follow up later on like, there was a doctor coming around who was on my diabetes, she was looking - I think she was a dietician - but she was also across how much exercise I was doing in the hospital

because they have you up walking very quickly. She ran through all that, she was excellent. She was great.

Facilitator: That was a nurse?

Interviewee: Yes that was a nurse.

Facilitator: That's good so you feel comfortable...

Interviewee: Doctors tell you nothing.

Facilitator: [Laughs] So how do you feel about the nurse assessing your mouth, looking at your mouth?

Interviewee: Oh well...

Facilitator: Doing a visual assessment.

Interviewee: They had a male nurse there that was looking at other things so a female or any other nurse looking at my mouth would be quite okay.

Facilitator: Okay so if a nurse gives you advice about, or refer you to a dentist would you attend, would you go?

Interviewee: Yeah I would.

Facilitator: Would you follow that advice? If there was a dental service offered free, for cardiac patients would you go, would you attend?

Interviewee: I probably would yes.

Facilitator: You would?

Interviewee: Yes. You said the magic word, free.

Facilitator: [Laughs] Exactly.

Interviewee: However I'm one of those people that if I go and I don't like the dentist - like I had a dentist once, I had him once, he had bad breath worse than mine. So that's it I just got up and walked out. Don't put up with that.

Facilitator: You were uncomfortable?

Interviewee: So if he's okay yes.

Facilitator: So when do you think is the best time to receive information about oral health? If you look at back from when you got sick to now, when do you think would be the best appropriate time for you to receive information about oral health?

Interviewee: To be quite honest any time between I had my turn and probably five days after the operation, we're talking several months, I wouldn't have been receptive.

Facilitator: You wouldn't?

Interviewee: Because I was too worried about everything else. So yeah, I'd say probably when you're in hospital, after a couple of days and you're getting about a bit better. You're not aching and paining as much, you're more receptive to listen - probably because you're bored lying there but, yes I think you're more receptive. From there up to the operation, I probably wouldn't have gone anywhere or had anything done, I probably wouldn't have listened - I would have listened but it just...

Facilitator: Yeah it wouldn't have.

Interviewee: ... it was all just…

Facilitator: What about during the rehabilitation program?

Interviewee: Yes, now would be probably a good time, yeah definitely.

Facilitator: You attended the session on Wednesday isn't it, yesterday there was a...

Interviewee: Nope.

Facilitator: Ah you didn't come, no that's fine [laughs] because they do education as well. Yes I was just wondering.

Interviewee: No I didn't come.

Facilitator: Do you feel nurses are sufficiently trained to give you information about oral health?

Interviewee: I don't see why they are not sufficiently trained; they're in all fields so, yeah.

Facilitator: Let's talk about your social support, which’s your support, socially you're married, you mentioned a wife before.

Interviewee: Yeah, my wife and two bludgers.

Facilitator: [Laughs].

Interviewee: A son and a daughter. One's a forensic scientist, that's working as a bum for (Name Omitted)., the other's got three degrees in electronics, he's currently working for (Name Omitted)., but whether he'll have a job

at the end of this year is, they don't know until everything's over. They get their diplomas and all the rest of it and then they'll know whether they've got an ongoing job.

Facilitator: So when you were in hospital and if you needed to go anywhere at the time, who's helping you at the time was it your wife?

Interviewee: Oh yeah.

Facilitator: She drives?

Interviewee: Oh yeah.

Facilitator: How do you make it to your appointments or come here, you come by, do you come by...

Interviewee: Come here?

Facilitator: ... public transport?

Interviewee: Public transport yeah. Primarily because the only thing the government's given me is one of those Seniors Cards. Which is the State Government anyway, and the bus goes past my door. I only have to walk down two houses to get on the bus, lets me out the front of the hospital.

Facilitator: That's good.

Interviewee: Couldn't be easier. It's $2.50 and on the one ticket I can catch another bus and another bus and a train, another bus. So yeah it's good value for money. I could drive in, but in the early days I found that, as soon as the six weeks is up, and Dr (Name Omitted) looked at me and said oh yes, it's knitting all back together, you should be able to drive from now on. Oh great I'm going driving. The first time I drove for a couple of hours then I got out of the car and I felt like someone had got a piece of broomstick, and just belted me all over the chest and the back. Just going from six weeks of not driving, to suddenly just holding arms out like that driving, just enough I thought, I think I better go to this rehab and [laughs] no more driving for a while.

Facilitator: Getting your muscles...

Interviewee: Yeah.

Facilitator: ... built up.

Interviewee: Yeah and I think the three years prior with the diabetes, I'd been running myself down, I was a 127 kilos and by the time the beginning of this year came, I was down to between a 100 and a 105. That's the reason originally they wouldn't diagnose me with diabetes. I kept saying to the doctor I said look, I'm losing weight. It's only when I went in and I said look I go to work, I work for 20 minutes, I'm dry, I get four ounces of chilled filtered water out of the fridge, I drink it, 20 minutes later I've got to run for a pee. Not stand there and think oh I might have a pee in a minute, I've got to be running to have a pee. Twenty minutes later I'm dry. I drink, 20 minutes later I piddle. Twenty minutes later. Oh he said oh, you might have diabetes. I've been telling you that for 12 months. So I changed doctors.

Yeah well I figured that it's getting close to the point where I'm going to retire, having a doctor at (Place Name Omitted), I had a company car see up until I finished work, I could drive to him anytime. Whereas the doctors in the Medical Centre at (Place Name Omitted) they're only 900 metres down the road. That's where I walk every day now anyway. So I go in and see Dr Jee, he is younger, he is more receptive. Dr (Name Omitted) didn't even have a computer; all his records were on bits of paper. So I thought it's time for an upgrade.

Facilitator: [Laughs].

Interviewee: As much as I like Dr (Name Omitted), but I tried to tell him I had diabetes and kept fobbing me off.

Facilitator: Well thank you. I just have the last question basically have you got any comments in relation to what we've been talking about and in relation to oral health and cardiovascular disease or any other questions?

Interviewee: Well I didn't even realise there was any join between oral health and cardiovascular. I thought it was all down to salt, too much salt, too much cholesterol but as I'm finding out now all my arteries have cholesterol in them, and then over the top of that, it's not salt, it's calcium, its calcification. I think that's what then affects the diabetes, because isn't diabetes the cells need insulin, to open the cell wall to allow the oxygen and the blood glucose to go through the cell wall into the cell, so the cells can then burn that to make your energy and what have you?

Facilitator: That's right.

Interviewee: The waste products, the carbon dioxide and water, comes out in your veins. Now if the lining on your artery has cholesterol and then a rock hard layer of calcium, how the hell does the stuff get through to the veins? So at night time when I go to bed my legs feel like they're absolutely full of maggots. Maggots crawling inside my legs and you just can't sleep. It's probably why every now and then I just can't get up in the morning I'm just so tired. But you're tossing and turning and when the sugar levels first came down, when I went on to the diabetic medicine, it seemed to improve my sleeping at night. But I think coronary heart disease and diabetes, I think they're linked and for some reason they kept telling me that oral health and diabetes was linked, so maybe it's linked to this too.

Facilitator: Yeah there is a link actually and that's why we're doing this study there's a strong relationship between poor oral health and it's just inflammation, it's to do with inflammation.

Interviewee: When you're saying inflammation do you mean like…

Facilitator: Inflammation in gums, the inflammation of the gums, or even a tooth.

Interviewee: Where a tooth becomes so inflamed it actually pushes down.

Facilitator: That's a condition, an oral condition but the inflammation is long term.

Interviewee: Yeah.

Facilitator: It can cause increased chances of getting arteriosclerosis so basically the plaques that form in the veins, which in turn can become - people may have recurrent strokes or recurrent cardio problems. They've found lot evidence that people that already have problems may have newer problems, recurrent problems. That's why we think that it's quite important that we give education to patients early. Especially patients that are being diagnosed with cardiac problems, because we know that there's are definite link there. All the organs are associated.

As you mentioned diabetes, this link you feel the diabetes is poorly controlled, you get bad oral health, the inflammation, problems with the gums and the teeth. If there's problems with the gums and the teeth the diabetes get bad control, so it's a reverse problem with diabetes. But with the heart that's what we know, so far. So we find it important to pass the information and hopefully do something to refer patients to a

dentist early enough and hopefully do it as a free service as a normal referral pathway for all cardio patients.

Interviewee: Oh right.

Facilitator: So that's basically it. Yeah.

Interviewee: You might like to also know that my mother's 92, has suffered from angina all her life. My father died at 75, which is about 10 years ago, he had cardiomyopathy, he had pacemakers and all sorts - but he had a heart attack in his 50s. I think that's what's caused the damage to the heart which is giving me cardiomyopathy and of course the dead spot's gotten bigger to the point where [unclear]. One thing he always complained about was aches and pains in the legs, but he had no diabetes. He lost lots and lots of weight, and I think that's a function between the brain and that. If your heart's not circulating enough blood and oxygen, you cut circulation to your extremities. So like my arms and legs, his really, he became really thin. His brain at the end - he was still sharp as a tack he was still very smart man but he just wasted away to nothing.

Facilitator: Possibly he had peripheral vascular disease. Some people can have it without being diabetic, [unclear] problems. Okay (Name Omitted) thank you very much for talking to us.

Interviewee: That's alright.

Facilitator: Talking to us, to me and everything to do for this, and hopefully something will come out of all this and we'll be able to develop a good program to prevent problems happening.

Interview 8

FILE DETAILS

Audio Length: *20 minutes*

Audio Quality: ☒ *High* ☐ *Average* ☐ *Low*

Number of Facilitators: *One*

Number of Interviewees: *One*

Difficult Interviewee Accents: ☐ *Yes* ☒ *No*

Other Comments: Interview in Arabic and English. Interviewee's daughter translated

---

Facilitator: Thank you very much for agreeing to participate in this interview

Interviewee: Okay

Facilitator: First I would like to ask you about the health of your mouth, gums and your teeth. How much do you know about the topic of your oral health or the health of your mouth and teeth in relation to your heart? Have you ever heard anything about that?

Interviewee: No..no [Interpreter in Arabic, Oh I know about it

[Interpreter in Arabic]

Interviewee: I know about it, yes

Facilitator: What do you know?

Interviewee: I know that anything in the heart. If I smoke or eat something wrong or too much eat I must clean the mouth. This is good for the health

Facilitator: For the health

Interviewee: And for the heart. I know about it

Facilitator: Good, good. And when did you hear about it? Did somebody tell you, did you read it somewhere?

Interviewee: They told me about it. (Cardiac Rehabilitation Nurses Names Omitted) (Cardiac Rehabilitation Nurse Name Omitted) tells me something special about the weight and I try to make it and (Cardiac Rehabilitation Nurse Name Omitted)…they are too good, they give me advices, I see is good for me...and you. You give me questions is good.

Facilitator: Basically you said that you heard about it. Do you know anything specific? How can anything in the mouth affect the heart, more specific information? Or just for the health in general?

Interviewee: No, just general

Facilitator: You said that you were told. Have you ever received anything written or on television or anywhere else?

Interviewee: General, yeah I know about it but let me tell you something about my teeth; before I came here I lived 5 years in (Country Name Omitted). My teeth in (Country Name Omitted) I make it before came here…I make it all; I paid money in (Country Name Omitted) I make it in doctor in (Country Name Omitted).

Facilitator: A dentist?

Interviewee: Yes. But something trouble about it now. Maybe I need to clean my teeth. Sometimes I smoke so I must clean my teeth sometimes. But now I have some trouble

Facilitator: So what is the trouble, what happens, what is the problem now?

Interviewee: Pain here sometimes. So I take an x ray, I go to my dentist; I take an x ray, trouble here. They want to pull out my tooth, only one, I refused

Facilitator: You don't want to?

Interviewee: No I don't want to pull out

Facilitator: Can they do treatment, can they treat it somehow?

Interviewee: No, generally is fine

Facilitator: So is not too bad?

Interviewee: No, is not too bad. My doctor tells me as you wish. If it hurts you I will take it off but I refused. Now I try to keep it like this

Facilitator: So what do you do to keep it good, what do you do to look after them?

Interviewee: I try to clean it, wash it and don't eat

Facilitator: Don't eat on that side?

Interviewee: Yes, eat on the other side

Facilitator: Do you floss as well?

Interviewee: Yes

Facilitator: That's good. You said you have a dentist. How often do you see your dentist?

Interviewee: When I need it I go

Facilitator: So when was the last time you went?

Interviewee: Maybe 2 months

Facilitator: That is when you said you had the pain

Interviewee: No pain I don't go [laughs] I don't need to go now. But sometimes I feel…sometimes

Facilitator: aha...aha. Obviously maintaining your oral health is important to you, I can see that. You also mentioned about your information.

So, would you like to receive more specific information about oral health and your heart. How can the heart be affected. Would you like some information about that?

Interviewee: No, I don't need it

Facilitator: You don't need it?

Interviewee: I know about it. I read about it in my country Iran. I have finished high school you see. I know about it

Facilitator: That is very good

Interviewee: I take my health now...you see me I am 71

Facilitator: Yes, you look well

[laughs]

Interviewee: You see I don't need any advice about this

Facilitator: Aha. That's fine

Interviewee: I take care of myself

Facilitator: I can see that you look after yourself

We talked about the dentist, sorry I want to make sure that we covered everything, you mentioned about your practices, and you told me a little bit about your experience with the dentist -he let you decide whether you wanted to have your tooth out or not. And you did mention that you had your work done before you came into Australia.

Interviewee: Yes

Facilitator: How long have you been in Australia?

Interviewee: One year

Facilitator: Ok, so you saw the dentist about a year ago. Did you need lot of treatment then? Did they put new teeth or filling?

Interviewee: Yeah, they put two here, one here and one here [pointing at his mouth]

Facilitator: New teeth or filling?

Interviewee: New teeth

Facilitator: That is good

Interviewee: I made it all before I came

Facilitator: Do you have any problems o difficulties looking after your mouth and teeth?

Interviewee: No, no trouble

Facilitator: And going to the dentist. Do you find there are any challenges going to the dentist?

Interviewee: Is no problem but I don’t try to go to the doctor

Facilitator: Why not?

Interviewee: waiting…pain. Look, between us I don’t like sitting and waiting for a doctor, any doctor. Especially here, waiting one hour, half hour. I have three doctors here: my GP, my back specialist, my heart specialist. So all the time I go to doctors. I must visit three doctors so I don’t like to make it four.

[laughs]

Facilitator: That makes sense to me. I get what you mean. So when you see your dentist do you have to wait long to see him?

Interviewee: They gave me a referral to go to another doctor. They told me “we can’t do it here in hospital; you must go to another doctor”

Facilitator: So you went to the dental service in the hospital?

Interviewee: When I went to the dental hospital in (Name Omitted) they sent me to another doctor [specialist dentist]. He said “sorry this is very difficult to me”. So I took the bill $300 and took it to the other doctor. They paid me in a Boucher

Facilitator: Did the hospital give you a Boucher?

Interviewee: Yes, I gave it to another doctor here in (Name Omitted), they take $300 and they do nothing

Facilitator: So, the hospital couldn’t do the treatment that you needed and sent you to somebody else and you used the same Boucher. You did not have to pay with your money

Interviewee: No, no, I don’t pay

Facilitator: Aha ok I understand that

Interviewee: I pay for my eyes

Facilitator: Pardon?

Interviewee: I pay money for my eyes

Facilitator: You pay for your eyes, so you also have an eye specialist. So another one… [laughs]. You mentioned about high pressure in your eyes so you get eye drops.

Interviewee: Yes, two eyes. I have the drops

Facilitator: Is it glaucoma?

Interviewee: No, pressure. High pressure in my eyes

Facilitator: How do you feel about the nurse in the cardiac rehabilitation, you know, when you went to see (Cardiac Rehabilitation Nurse Name Omitted). How do you feel about the nurse talking about your oral health, the dentist, checking your mouth and assessing you when you see her? Do you feel ok with that, are you confortable with that?

Interviewee: Yeah, yeah. I feel good. Doesn’t matter, ok. No problem

Facilitator: No problem. So if she [rehabilitation nurse] gives you information checks your mouth and asks you questions…

Interviewee: I need that, I need that

Facilitator: You need that?

Interviewee: They check me I am glad for it. That they check me, ok

Facilitator: Can you tell me what kind of support you think you need to help you seeing a dentist regularly for example?

Interviewee: From the dentist?

Facilitator: From us, from the nurses, the health systems from us so we can support patients like you, to make it easier for you

Interviewee: For now is ok, from you, from (Cardiac Rehabilitation Nurse Name Omitted), (Cardiac Rehabilitation Nurse Name Omitted) and the hospital with my nurse: good manners, good what they are teaching about is all good. I can't believe it. Because in my country is not like this

Facilitator: Is it different?

Interviewee: It is different. Is all good. But I tell you about another side. If I tell you will answer this is not my job

Facilitator: Ok I understand

Interviewee: I know what you job is about. Your job is ok. I say the hospital the rehabilitation nurse is very kind. I tell the nurse when I finished "you are like my daughters", is good.

Facilitator: Is good that you feel that way. You need to feel comfortable, especially when you…

Interviewee: I am very glad about it. My nurse, are you a nurse? I am very, very glad about your attention, all. I am astonished about this. I didn't look about this before, believe me. After they did the operation in the hospital (Place Name Omitted). I sleep one day, they try me gently, even to take a shower. I told all my friends here about the shower. In (Country Name Omitted) no shower, no nothing but here, I am very glad about it.

Facilitator: Yes, the conditions are a lot better in Australia

Interviewee: I'm very glad about it.

Facilitator: You are happy about nurses giving you advice. Do you think nurses have enough knowledge to tell you information about your health?

Interviewee: Yeah, yeah. Even in (Country Name Omitted). Before I take the medicine for my back, capsules tablets, (Cardiac Rehabilitation Nurse Name Omitted) told me this is bad for you, this is bad for your heart. Stop it, don't take it so I stopped it and I told my doctor. Another one, (Cardiac Rehabilitation Nurse Name Omitted) gave me a message to give to my specialist doctor about the medicine. I took the message to him.

Facilitator: So the nurses told you about it. Is right, some medicines can affect you heart or heart medications. It is important that you are informed

Interviewee: They are very intelligent about the medicines...very clever. Yes, you are all very good. You are too

Facilitator: Me? I am just doing the study but if I can help you with anything I will. Now, I can see that you have very good social support. You support is your family? When you have appointments or for your surgery or in hospital

Interviewee's Daughter: Yes we don't leave him. We stay with him until he is finished and then we go home.

Interviewee: They come, my wife and my daughters. They cry...they cry

Facilitator: Of course, you are their dad. I would cry [laughts]. Is there any questions or comments you want to make about what we are planning to do with this program, basically get the nurses to inform about the mouth and teeth as part of the normal information given to patients with heart problems do an assessment and refer them to the dentist if necessary. Are you happy with that, do you think is a good idea, what are you comments?

Interviewee: In the hospital?

Facilitator: When you have your heart assess they would talk about your mouth as well as part of the whole package .and if you need help they would send you to the dentist straight away. Because at the moment in Australia it does not happen, Cardiac patients are not referred to the dentist commonly so we are trying to incorporate it into their practice

Interviewee: Will you give me an order for the dentist?

Facilitator: That is the plan, it is not happening yet. We want to make it part of the system

Interviewee: No now? After

Facilitator: After, yes

Interviewee: It doesn't matter

Facilitator: At the moment I am collecting information, what you tell me, what other patients tell me, what nurses say, we will put it all together; we will present it to the government and say this is what we found

Interviewee: No problem

Facilitator: So do you have any comments?

Interviewee: About all?

Facilitator: Yes

Interviewee: I can't believe here is good guidance in Australia, I don't believe it, believe me. Thank you, thanks to all of you. Thank the government, all because they treat me like a king! [laughs]

Facilitator: Thank you very much for the interview and your daughter for helping with interpreting

Interview 9

<u>FILE DETAILS</u>

Audio Length: *22 minutes*

Audio Quality: ☒ *High* ☐ *Average* ☐ *Low*

Number of Facilitators: *One*

Number of Interviewees: *One*

Difficult Interviewee Accents: ☐ *Yes* ☒ *No*

Other Comments:

Facilitator: Thank you for agreeing to participate in this talk

To start the conversation I would like to talk about your oral health or the health of your teeth, gum and mouth in relation to cardiovascular disease or your heart condition. Can you tell me what do you know about the topic of oral health in relation to your heart?

Interviewee: I don't know anything about it but now that I went to the dentist the other day he pointed out about my gums having a problem but he actually has being telling me for quite some time that I need to use anaesthetic to get rid of the thing to clean inside. Because is painful I just feel it is hard to do and I just ignore it, for that reason I don't even want to see the dentist and I try to use electric toothbrush but actually when I went to see the dentist the other day he said that my gum raised up.

Facilitator: Are your gums swollen?

Interviewee: Yes, I was drawn to my attention; I was having gum problems a year ago went I was having chest pain all the time and I went to see the dentist and he said “oh, you’ve got a big problem” with my mouth and “we really need to do something” and then I bought nice toothpaste that is good to clean up the mouth after drinking alcohol and I do pay attention and it did get better and then when I don’t pay much attention probably that risked me and I had a big heart attack that I had to call the ambulance. So I realised when I recall what I did. When I go to (Country Name Omitted) you kind of travelling and you don’t look after it that well when you are in a hurry to do things and you forget to brush your teeth at night and staff. When you are travelling you ignore it a bit and it became a bigger problem for me and my heart

Facilitator: So it wasn’t your priority at the time, you were busy doing other things

Interviewee: Yes, you didn’t realise but now when you look back, and now you say that it has…the dentist actually told me because I told him I had an operation he said that “I don’t know what it is, they think that there something linked with your heart” and then straight away it drew to my attention that yes, yes…

Facilitator: The dentist mentioned there is something to do with the heart. Did the dentist tell you exactly?

Interviewee: He told me as well this time but he didn’t tell me last time

Facilitator: What did he say?

Interviewee: This dentist told me that “I don’t know what it is, the studies found that there is some linkage with heart attack when you teeth are not clean because this happened to his grandma” and he said “there is positive linkage” he doesn’t know what it is but the study that told him…straight away I’m like “oh no…” yeah, when I think and look back. I agree with him so he tough me how to floss my teeth, how to clean up and I think I want to do the proper clean up when my heart is healed up because is too fresh. Because after he cleaned my teeth that day I was exhausted, very tiring. I haven’t being tired for a while, when I healed up properly and that day when he did something to my teeth I think my whole body was very exhausted because I had the surgery

Facilitator: After the surgery you did that

Interviewee: Yes, because probably still due to my heart I was very tired, extremely tired that time. I have being recovering well I haven't being that tired but I thought it was important because he cleaned my teeth very properly and then after that day I feel more healthy, even in my heart

Facilitator: So you have been to the dentist since the operation, when was this?

Interviewee: I haven't been to the dentist since the operation that is why I haven't being…

Facilitator: Was it before the operation? You said that they cleaned your teeth…

Interviewee: Oh, yes after the operation I went to the dentist. It was a couple of weeks ago

Facilitator: And before the operation?

Interviewee: I haven't. For a year I haven't seeing the dentist because I think I ignored it but a year ago or two the dentist warned me I have gum disease

Facilitator: Right

Interviewee: I have gum disease, I don't want to hear

Facilitator: So, you ignored that

Interviewee: I ignored it but I cleaned my teeth better but after a while I forgot you know. Only when he warned me I did something then I went back to the normal thing

Facilitator: What prompt you to go to the dentist? Did you decide yourself?

Interviewee: No, I was walking on the street and bump into my friend. He is a dentist and he dragged me to his dentist…I'm like ok, alright I went because I thought I haven't clean my teeth for a while and then I did it. I was hesitant because it was just after the operation and then I said: "Don't give me any pain, just make it whiter" and then he pointed out that I've got so many problems and then I realised "oh shit it could be something to do with my heart as well!"

Facilitator: So your friend took you to the dentist. Your friend is a dentist

Interviewee: I didn't used him I said "give me a good dentist" to go to in his surgery. He's got a few and then he asked me to see this guy I don't know the name but he said that his grandmother has the same problem because I

told him my heart things and told him to be gentle I just want him…but he did not clean my teeth very well but I was really tired

Facilitator: Because you had the surgery not long before

Interviewee: Yeah exhausted, really, really tired. I thought that “why cleaning my teeth cause me to be so exhausted” but after that day I feel that helped with my recovery as well. Because he said “your gum has some lumps but after that he cleaned properly and my gum healed up

Facilitator: Since you’ve seen the dentist are you looking after your teeth more?

Interviewee: I floss my teeth everyday now

Facilitator: Every day, and what else do you do to look after your mouth?

Interviewee: I’m starting to use the electric [toothbrush] very well, change every month. Also use the mouthwash

Facilitator: So you brush with the electric toothbrush that is good. How often do you do that?

Interviewee: Twice per day. What the dentist recommends. At first he said “you’ve got to do it every day”. Now I’m on sick leave I have more time so I do it properly

Facilitator: Ok, that’s good. That’s very good. It is very good information what you are giving me. Obviously it is important for you to maintain your oral health. The dentist gave you information; did they give you anything written or just verbal information?

Interviewee: Verbal

Facilitator: You mentioned you haven’t heard anything about it before

Interviewee: No, only this time when I told him. He said “any history?” and I said “I just had open heart surgery” and then he asked me more question and went “hang on, hang on, hang on…” and then he said that his grandma had the same thing. She had gum disease as well and he said “now, you’ve got to floss your teeth every day” and he tough me, he spend actually quite a long time to educate me because of it. Yeah, only he brought it to my attention, never before. But before the dentist told me my gum had disease that I needed to do it and I just quickly run away, I was scare of the operation.

Facilitator: Oh, you were scare to see the dentist and do the procedure

Interviewee: The procedure was scary; he said I needed to get rid of the gum to clean inside. I need to put this up

Facilitator: The gum up, is that what you are saying?

Interviewee: Yes, yes. Because it is very scary, in the whole mouth. I need to cut the gum and clean. He said he would do it four different times other ways my whole mouth will be painful

Facilitator: So you need to go back to the dentist again?

Interviewee: Yes, but not now because is after the operation. He said that is not very urgent but I've got to do it sometime because it will release the bacteria inside

Facilitator: Yes, but you are doing the right thing now. You are cleaning your teeth

Interviewee: I clean properly. I made an appointment three months later when my heart heals up to do it one by one, quarterly

Facilitator: You mentioned that you got information verbally, would you like to receive information in any other way?

Interviewee: Sure, because now that it was drawn to my attention it is causing big problems; I need to deal with it

Facilitator: In which way would you like the information to be given to you?

Interviewee: Whatever, email or mail is all good. E mail I have a look at

Facilitator: What about leaflets or something like that?

Interviewee: Yes, actually information. I think is good to draw it to people's attention to prevent…Prevention is better that cure. Cure is very expensive, scary and physically torture

Facilitator: That right. What about receiving information from the nurses in a talk like today [education day for cardiac rehabilitation patients]. In the cardiac setting like today when (Cardiac Rehabilitation Nurse Name Omitted) was giving a talk about risk factors

Interviewee: Yes, we can find out all about it. What risk factors can cause because it was never drawn to my attention. If it was drown to my attention I probably would've had a proper clean up a long time ago to prevent this from happening

Facilitator: If the information was given top you. Would you prefer it in a different language? You said that you speak Mandarin and Cantonese. Or is English ok with you?

Interviewee: Is ok I understand English. English is ok

Facilitator: We talked about your dental health. Do you feel your mouth is ok now, your teeth and your gums?

Interviewee: I think it healed up because I look after it

Facilitator: That is very good. So you haven't got any concerns about it now?

Interviewee: Not right now, no but I think I need to clean it up properly

Facilitator: With the dentist?

Interviewee: Yeah

Facilitator: Do another clean up you think?

Interviewee: Yeah, but it is a procedure I think

Facilitator: Yeah, the dentist will have to tell you that, how he is going to do it. Do you think you experience was good with the dentist?

Interviewee: It was ok, yeah, yeah

Facilitator: You did mention that you were a bit scared before

Interviewee: Before he didn't point out that it can lead to my heart. Actually my heart had frequent chest pain all the time and he said that the whole mouth had a lot of bacteria and big problem, big problem but that was the other dentist. He did tell me anything with my heart so I just ignored him and I did not go to see the dentist for a year and a half but then I took action, tried to clean it properly but they said is unclean able because there is a stone inside my gum, you can't actually clean, you need a procedure. I do know how it got in but it went in…

Facilitator: What do you feel about having an assessment or looking at your oral health when you come to see the cardiac nurse, for example when they do the assessment at the beginning when they take your weight and talk about your health and risk factors, how do you think about them talking about your oral health, looking at your mouth and checking that is all ok and send you to the dentist if you need to go?

Interviewee: Ok, sure I need because now is being drawn to my attention, I need to pay attention to it and see what else I can do because I think the dentist only looks after your oral health is not their responsibility to look after your heart but this guy was pointing it out because his grandma having the same issue and he draw to my attention. It was accidental, if his grandmother was not…I don't think he would have tell me the story as well. I've seen a lot of other dentists they always said there is some stone inside it is not like a major risk. It is good to do it, to clean it up

Facilitator: So you think is a good idea that they can talk to you in this setting with (Cardiac Rehabilitation Nurse Name Omitted) or with the cardiac people?

Interviewee: Now is drawn to my attention, I need to do something

Facilitator: What kind of support do you think you need to help you attending your oral health, the dentist more often? Do you think you need some help or are you confortable?

Interviewee: I don't really know now that it was drew to my attention, to pay more attention but you are scared of the procedure but after I had the heart surgery I was more scared of the heart that is nothing compared but you are still scared

Facilitator: Of course, it is scary for many people. So do you think you need some support to help you making the process easier to go to the dentist or are you ok with what you have now?

Interviewee: Yeah, I don't know what to say

Facilitator: Is ok

Interviewee: But if people don't understand that if you do not brush your teeth you are going to have a heart attack they will be more motivated. I would've not done it if my friend did not dragged me and force me into seeing the dentist. I was probably was not to see him for a long time. Now I see was very good because my heart probably would have taken longer to heal up if I didn't clean up properly…and pay attention to. But now even you giving me this talk and directing me to pay more attention to it I am willing to participate in any more appointments and support in this area.

Facilitator: That is beautiful, thank you

Interviewee: You pay attention, not playing with pain. If you are not paying attention there is more pain. I came as a big shock. It was an emergency and all of the sudden this was too much for me to handle. Now I need to pay attention to it

Facilitator: To your health, that's right. Do you have social support or network? I noticed that you a very independent

Interviewee: To be honest my family is in (Country Name Omitted), I'm single and I actually don't talk to my friends about my problem. I handle it myself but I handle it quite well but this support is good for me I only pop within this, I don't think my friends will help me properly, they just give me wrong advice and…but I do play sport with them. I have a soccer team, a badminton team, social team, jog team. I don't talk to them about my problems because they would just give me wrong advice or I feel they would look down on me because…

Facilitator: It is a different kind of group isn't it if you play sport you do not want to talk about your health; you may want to talk about other things I imagine

Interviewee: Even though they called for Christmas I didn't tell them I had an operation

Facilitator: Oh really. Do you live by yourself? When you had the surgery, who helped you, did somebody came…

Interviewee: My sister did come to visit but I feel like she didn't understand me. I tried to tell her not to come but she kept coming because she was worried

Facilitator: Of course, she is family. Does she live here in Sydney?

Interviewee: Oh, she is a best friend, not even a sister but she lives in Sydney, she provided physical help but when I got home I just tried to do everything myself

Facilitator: You are very independent

Interviewee: Yes, quite independent

Facilitator: Do you drive to come here?

Interviewee: Yes I drive

Facilitator: And do you normally work, did you work before the surgery?

Interviewee: I work

Facilitator: Are you working now? Are you back at work?

Interviewee: Not yet, I am back to work in February. They starting to send me meeting requests I am feeling it

Facilitator: Say I am on sick leave now

Interviewee: I actually don’t want to read it. They started to send me manager meeting requests and all that, I’m like ohhh I don’t really want to work anymore but I have to face it in a couple of weeks, yeah

Facilitator: I have a couple of more questions, almost done. Would you follow the nurse’s advice to see a dentist if they tell you to do that?

Interviewee: Yes

Facilitator: And do you think the nurses have enough preparation to talk to you about oral health or the health of your mouth and teeth. Do you think that the nurses, somebody like the cardiac rehabilitation nurse, do you feel she is prepared enough to tell you and enough knowledge?

Interviewee: I found it is very helpful with this (Hospital Name Omitted) program because I try to Google everything and my friends give me wrong advice and this one, people have professional help and they understand what they are doing and their advice is very professional like my friend from soccer and my sister, they don’t really know this things they tell you a lot of advice there wrong but. Sometimes is frustrated because you are in pain and staff so I just stopped talking to them about my problem because I don’t think is helpful but their support their worry is genuine, that helps. They just need to be a friend and not give me advice on these things

Facilitator: I understand. Do you have any comments or suggestions about what we talked about, everything that we said today?

Interviewee: I think this study need to tell the dentist. The dentist needs to educate their patients as well. I have being seeing the dentist every now and then and the dentist all informed me about my gum disease but if he said “oh if you don’t do it you will have a heart attack because this is your age, your got to pay attention” I could’ve done the procedure beforehand because it has been a long time. It is firstly expensive, it cost me money it is private and did not want to do it. Secondly it is painful and thirdly “oh…some stone inside my gum is not a big deal, just

leave it, it has been there for ten years it probably can stay there for another ten years". You cannot ignore it. It is money, you feel I don't have extra a few hundred dollars to do this thing, it is painful, if it is inside for long time let it be inside for another ten years. I tried to ignore it. Is what it is, is all about that. Until this big open heart operation, is a wakeup call but you would've want the warning before this wakeup call. You would've want to do it before hand rather than afterwards

Facilitator: Yes that's right, looking back

Interviewee: It's too much to handle

Facilitator: But is a new beginning now

Interviewee: Is a new beginning yeah. It's good to be aware of it before but I wasn't. Is all I can say

Facilitator: Thank you very much, you given us lot of input. We are going to take all into consideration when we develop this program

Interviewee: Yes, ok. Thank you ok

Interview 10

<u>FILE DETAILS</u>

Audio Length: *18 minutes*

Audio Quality: ☒ *High* ☐ *Average* ☐ *Low*

Number of Facilitators: *One*

Number of Interviewees: *One*

Difficult Interviewee Accents: ☐ *Yes* ☒ *No*

Other Comments:

Facilitator: Thank you for agreeing to the interview with me today

Interviewee: You are welcomed

Facilitator: Ok, we are going to go straight into the questions. Can you tell me what do you know about oral health or the health of your mouth, gums and teeth in relation to your heart?

Interviewee: Absolutely nothing

Facilitator: Never heard anything about it?

Interviewee: No

Facilitator: Ok, I imagine that the first time you heard something about it is when I approach you?

Interviewee: True, that's why I decided to participate

Facilitator: Thank you for participating. Is your oral health important for you?

Interviewee: I think so; I mean the only oral health that I use is…I have a mouth wash that I use regularly which I have done for many years but I don't have my own teeth

Facilitator: Alright, so you have dentures?

Interviewee: That's right but I know that it can get swollen at times

Facilitator: You are talking about the gums?

Interviewee: Yes so that's when they are not healthy

Facilitator: Have you had any oral issues or have you got any problems at the moment?

Interviewee: I haven't had it so bad that I had to go to anybody or do anything about it

Facilitator: Alright. So how long have you had your dentures for? I you don't mind me asking

Interviewee: Oh…I had dentures since I was 28, I am 78 now

Facilitator: A long time. So, your mouth has never been a huge problem?

Interviewee: No

Facilitator: Do you see a dentist?

Interviewee: As I said now and again I get swelling and I worry about it but then it subsides and everything is fine

Facilitator: So you don't have to see a dentist?

Interviewee: No, I've never being to a dentist since. I had my teeth out, don't check them. Apart from getting new mouldings done

Facilitator: Ok

Interviewee: And these ones I have for quite some time they are overdue for replacement. They are in my bucket list of dollars…

Facilitator: Is expensive isn't it

Interviewee: That's right

Facilitator: You mentioned that you haven't heard of this subject before. Would you like to receive information about it?

Interviewee: Oh yes I would

Facilitator: And if there was information how would you like it delivered? Part of what we are doing is to develop information for patients because there is nothing much in Australia at the moment, so how would you like the information given to you, in which form?

Interviewee: You can send it in written form, I'm happy with that

Facilitator: You are talking about a leaflet or a letter or something like that?

Interviewee: Oh I see. Whichever turns out to be the best one I mean if they decide to have leaflets and provide the information that way

Facilitator: Which information do you read more? Which sticks to you mind more when you get information about let's say not smoking or health information in general, which way do you prefer. Which do you notice more when you receive it?

Interviewee: Well, in terms for myself I prefer to have something for me, written, correspondence, yes

Facilitator: Do you have access to internet? Do you work with the internet much like e mails?

Interviewee: I have access to it but don't use it or anything

Facilitator: What about television, advertisement that way?

Interviewee: Oh yes, television alright. I watch television

Facilitator: That's good. I am seeing which way is going to cater for more people

Interviewee: I am happy as long as I get something because I really want to know about oral health and what I can do to help protect myself

Facilitator: aha

Interviewee: From further heart attacks

Facilitator: And I will talk with you about that when we finish the interview and give you more details. So, we talk about your oral health and you said that you don't have any current concerns

Interviewee: No, well I have some swelling in my gum down here last week for a couple of days

Facilitator: And then?

Interviewee: And then it went

Facilitator: So when that happens, what do you do? Do you do anything when it gets swollen?

Interviewee: No, I just [moving her tongue around the mouth] I do a little exercise with my tongue [laughs]

Facilitator: ok [laughs]. You said that you don't see a dentist. Do you remember the last time you saw a dentist?

Interviewee: The only reason I saw a dentist after I had my teeth out was to get new teeth, replacement teeth. So there was no other reason for me to see him and that would have been about 20 years ago. As I said these are long overdue for replacement

Facilitator: Ok, are you planning to do it soon?

Interviewee: Is all go to do with cost I don't know how much it is but I think is quite expensive here and they don't subsidise it like in (Country Name Omitted). There's a subsidy that is quite large

Facilitator: So they subsidise it in (Country Name Omitted)

Interviewee: Yes

Facilitator: Oh they do

Interviewee: As long as you are a pensioner or unemployed or whatever

Facilitator: I believe that they do subsidise it here

Interviewee: I haven't being able to found anybody. I mean I've seen something written on forms I've got from Centrelink but I haven't being able to find anybody. I was living in (State Name Omitted) for a while and the doctors, it looks like they have thrown that out

Facilitator: Actually I've got some leaflets about the public dental service. I have one so you may want to call them and find out so you can see. I know

they did it for my parents bit I am not sure if they still do it now, they got dentures but I am not sure how it works not but I will give you the information ok?

Interviewee: Thank you

Facilitator: What are your views about including oral health information in the cardiac setting while you doing cardiac rehabilitation?

Interviewee: I think that is a good idea I mean because people are here for cardiac reasons and that is one of the causes for heart attacks

Facilitator: One risk factor

Interviewee: yes, definitely should be included

Facilitator: How do you feel talking to the nurse, the physiotherapy or somebody in the cardiac setting about your oral health and them giving you education, assessing you or referring you if you need it?

Interviewee: Is fine

Facilitator: You feel fine with that?

Interviewee: Yes

Facilitator: So you are comfortable with the nurse doing it

Interviewee: Yes

Facilitator: Do you feel the nurse is well prepared to actually give you information

Interviewee: oh, I don't know, that is up to the people who are doing it, I haven't seen anybody ever

Facilitator: ok. When do you think is the best time to receive information about oral health? If you think of your journey through the hospital when you came to have your surgery and then you came to do rehabilitation. When do you think is the best time to get information?

Interviewee: I think when I am leaving the hospital; we should definitely get something because when I left (Hospital Name Omitted) I didn't get anything right there

Facilitator: Are you talking about (Hospital Name Omitted)

Interviewee: No, (Hospital Name Omitted) in (State Name Omitted). There is where I had my operation

Facilitator: You think that when you leave the hospital is the best time

Interviewee: mm, yes. Well I mean they can do it at the beginning but usually at the beginning you are concern about what is happening to you

Facilitator: That's right

Interviewee: yes so that becomes the least of your priorities so at the end...that is another risk factor to avoid. By doing this plus that or whatever, you take measures…

Facilitator: So what do you think during the time of rehabilitation such as when you attended the talk last week [cardiac education day]

Interviewee: That was good, I really enjoyed that. That made up my mind to be part of it because before that I didn't think to be part of it. Even though I didn't know anything about it but then when I listened I said right

Facilitator: So you think this is a good time as well when you have the talks during rehabilitation

Interviewee: Yes, that's right

Facilitator: You can be informed at that time

Interviewee: Yes, because all the talks that we have relate to out cardiac health and this one doesn't, is missing

Facilitator: Yes that is the idea, we want to introduce it. Can you tell me what sort of support do you need to be able to actually look after you oral health better if you think it needs to improve? You mentioned before the cost

Interviewee: That's right. Cost is a definitive factor for me. That's why I said I am hoping to get new teeth and I could never be without my teeth

Facilitator: Of course

Interviewee: To me I'm sure is going to cost over $2000. I don't know how much but I'm sure it is and so, you know, that's a lot of money so that's definitely yeah

Facilitator: A major factor. You did mention that there is a subsidy in (Country Name Omitted)

Interviewee: Yes, that's right. The first time when I had my teeth done when they took them out they had healthcare but then you need an x ray of your teeth

out cost so much and it cost so much for you replacement of the new ones but after that it was all subsidised it was quite cheap

Facilitator: Yes, and then is cheaper because you don't have to see a dentist that often, if everything is ok, is it?

Interviewee: Well, you wouldn't [laughs] I'm long overdue

Facilitator: A few years [laughs]. But you don't have any issues eating or anything at the moment, do you?

Interviewee: Well my eating issues have to do with my health, that's why I lost so much weight but is all to do with my hyperthyroidism that I've got which is being treated for right now

Facilitator: Oh, that is another problem with your health. You said that you are comfortable with nurses giving you advice and you will follow the nurses' advice if they told you to go to the dentist or refer you

Interviewee: Yes I would

Facilitator: We sort of talk a bit about your social support before. Do you get support going around and getting to places, do you get much support?

Interviewee: Well, I haven't being going because I don't know the area and I don't want to use taxis because I thought they I too dear so the only place that I have being to is (Hospital Name Omitted) when I had to go for my hyperthyroidism last week with the specialist

Facilitator: Did you use public transport for that or…

Interviewee: I used a taxi. I don't know how to get anywhere I get lost. I don't know I get lost. It's not point for me going out there

Facilitator: So you go exactly where you need to go

Interviewee: I get a taxi. Anyway the other day I was explaining to the nurse doing total health with my doctor about my difficulties coming here [cardiac rehabilitation] with the heat and the rain and she said well that is considered a medical appointment so we've got someone that you can contact and they can arrange to pick you up and take you to medical appointments which is brilliant for me to go to (Hospital Name Omitted) and (Hospital Name Omitted) for my heart

Facilitator: So they can organise hospital transport

Interviewee: Is not hospital transport is private, sure they must be subsidised by somebody

Facilitator: That’s good

Interviewee: Well I rang them up today and you have to give notice so I won’t be able to use it for here because the only time I need it is when is really hot or when is wet

Facilitator: So it won’t be a regular thing

Interviewee: No, just have to be for all my specialist appointments that I go to, but that’s good because is so far and so expensive

Facilitator: So you need to give some notice

Interviewee: I have to give a week’s notice to pick me up. They obviously have a limited service because when I rang up they say my doctor supposed to register me online so then I said “oh you need to register online, leave it to you” [laughs]

Facilitator: So, how did you make it here today?

Interviewee: I always catch the bus to (Street Name Omitted) Street from (Street Name Omitted) and then I catch another bus to here

Facilitator: So you are ok to come to (Hospital Name Omitted)?

Interviewee: Well, what’s getting me is the heat I can’t take heat

Facilitator: And it has being quite hot lately

Interviewee: Too much so it has being nice in here the last few days

Facilitator: I think we are getting to the end. Have you got any suggestions or comments you want to make in relation to what we are doing and what we are talking about?

Interviewee: Nothing that I can think of that you can do but I am really pleased that you are doing what you are doing and, as you said, there is nothing in Australia. I never hear of it in (Country Name Omitted) apart from the fact that we get subsidised to get our teeth replaced at the dentist

Facilitator: And that is what we are planning to do. Basically develop a program to train nurses in the cardiac setting to give you that education and hopefully develop some education tools and resources for the patients and educate the nurses and the team to be able to do it to patients.

	Incorporate oral health assessment and referrals…and send patients to the dentists if necessary, and be subsidised under Medicare
Interviewee:	Yes, that would be really good. Do you know of anything I could do now to improve my health
Facilitator:	yeas, basically what you are doing you know rinsing your mouth regularly, keeping your dentures clean and that they feel properly as well
Interviewee:	That's the problem, they don't fit properly so that's what I said is overdue
Facilitator:	I'm going to stop this and we can talk a bit more. Thank you for the interview it has being nice. I don't want to cut on your lunch…
Interviewee:	Oh I forgot all about it…

Interview 11

FILE DETAILS

Audio Length: *9 minutes*

Audio Quality: ☒ *High* ☐ *Average* ☐ *Low*

Number of Facilitators: *One*

Number of Interviewees: *One*

Difficult Interviewee Accents: ☐ *Yes* ☒ *No*

Other Comments:

Facilitator:	Thank you very much for your participation
Interviewee:	Pleasure
Facilitator:	Can you tell me what do you know about oral health or the health of your gums, teeth and mouth in relation to your heart?
Interviewee:	I am aware. When I go to my local dentist he does have sign up in his reception saying that there is a link. So I was aware that there was a link. I didn't understand the details or any more than that
Facilitator:	Have you spoken to him about it or anybody else

Interviewee: No really, no

Facilitator: So, you only saw that sign there

Interviewee: Yes. I guess I look after my...I go once every six months to the dentist. So I look after my…I do as much as I can to with my teeth I guess so there's probably not much more I can do

Facilitator: Yes, if you go to the dentist. And how do you look after your mouth?

Interviewee: I do daily brushing, I try and floss but with mixed success you know, sort of sometimes I go well other times I don't. But I do make sure that I clean my teeth regularly

Facilitator: Ok, that's good you said once per day

Interviewee: Twice per day in the morning and in the evening

Facilitator: Since you have being diagnosed with a cardiac condition and through being an inpatient and now in rehabilitation, have you heard anything about oral health? Apart from the study we are doing now

Interviewee: Not really no

Facilitator: Have you got any oral health complains at the moment, any issues that you may be worrying about?

Interviewee: No, no is all good

Facilitator: That's very good. So obviously maintaining your oral health is important to you

Interviewee: Yes very much so

Facilitator: Since there is not information that you have received, would you like to get some information about the link about oral health and cardiovascular disease

Interviewee: Yes I am very happy to receive some information

Facilitator: And which form you think would be the best way of receiving information?

Interviewee: Probably mail so I can sit down and read it rather than an email, I receive a lot of emails. And post it to my home I think is probably better or through my dentist would be the other option

Facilitator: Through you dentist to be send to you home. Are you thinking about some leaflets or information…

Interviewee: Leaflets. That's all I'm thinking off. Just a brief two or three pages on what are the relevant issues are so I can sit down and have a read of it over a coffee

Facilitator: Nice. And when do you think is the best time to get that information if the information was given in the cardiac setting either as an inpatient or outpatient. When do you think is the best time to receive information about your oral health?

Interviewee: I think about a month after all the major face started because I think if you did it too early there is too much information and I think, you know, about a moth people start to settle and listen and actively seek information so what I though one to two months after

Facilitator: After being discharged home?

Interviewee: Yes

Facilitator: So what about in this setting, cardiac rehab?

Interviewee: Perfect

Facilitator: Perfect timing?

Interviewee: Perfect timing

Facilitator: I notice that they [cardiac rehabilitation team] do weekly talks and you have different talks

Interviewee: Yes, that would be a good time to have a talk on this and leave us with some information to take home

Facilitator: That's good. Now, you said that you see the dentist every six moths

Interviewee: Yes

Facilitator: So you recently have seen your dentist?

Interviewee: Yes

Facilitator: Do you have any problems or issues when you go to the dentist? Some people mention pain or fear. Have you had any experience…

Interviewee: I'm very lucky I never had a filling or a cavity so no. I have a good dentist that I have being seeing for a long, long time and once I don't

enjoy going to the dentist I don't think everybody does, it is part of my lifestyle

Facilitator: That's good. Do you face any challenges looking after your oral health or seeing a dentist or professional?

Interviewee: No really, no. It's all part of my yearly sort of program

Facilitator: That's good. For you, health is a priority I imagine

Interviewee: Yes, yes it is. I've being brought up that way from, you know, a child, you take proactive care of your teeth

Facilitator: That's right, that's good. You have mentioned the rehabilitation setting is the best to get information. How do you feel about the nurse giving you information, assessing you and looking at your mouth and you oral health, asking you questions about it and referring you to a dentist?

Interviewee: Yes, look I am for that but I wouldn't because I have my own dentist, I see no reason to go to any other dentist. I am very happy with that dentist so…

Facilitator: yes. I guess the question was directed at nurses doing the assessment and if they find any problems advising you to see you own dentist

Interviewee: Yes that's fine

Facilitator: So do you feel comfortable with the nurses providing that advise and information?

Interviewee: Yes I do

Facilitator: And assessing the mouth or your oral health

Interviewee: Yes, no harm whatsoever

Facilitator: So you are comfortable with the nurses doing it

Interviewee: Very much so they are excellent

Facilitator: You feel that nurses are prepared enough to do it

Interviewee: I think so, yes

Facilitator: Ok, that's good. Do you feel that you need any support to continue to look after your oral health?

Interviewee: No

Facilitator: I can see that you are very independent. Do you have much social support, do you need any support with your everyday care, going to the doctor, coming here

Interviewee: I have a wife and children so I have a good family structure. If anything goes wrong I'm supported at home

Facilitator: That's good, and you said that you work?

Interviewee: Yes, play football

Facilitator: Do you drive?

Interviewee: Yes, I drive

Facilitator: So you don't have any issues accessing, coming and going

Interviewee: No but I generally use public transport most of the time and I find that very efficient, you know and I can walk I suppose to walk as part of this program [cardiac rehabilitation] so I would rather walk than catch public transport or drive specially in that city you know parking is terrible and expensive

Facilitator: I think we are almost done. Do you have any other comments or suggestions in relation to what we are trying to do with this program?

Interviewee: I think is a great idea, I fully support it. Very good

Facilitator: Thank you very much

Interviewee: Pleasure, alright that was nice and easy. Thank you and good luck with the study

Facilitator: Thank you and good luck with the exercise

Interview 12

<u>FILE DETAILS</u>

Audio Length: *9 minutes*

Audio Quality: ☒ *High* ☐ *Average* ☐ *Low*

Number of Facilitators: *One*

Number of Interviewees: *One*

Difficult Interviewee Accents: ☐ *Yes* ☒ *No*

Other Comments:

Facilitator: Thank you very much. I am going to go straight into the questions. Can you tell me if you've ever heard anything about your oral health or the health of your mouth, teeth and gums in relation to your cardiac health in the past?

Interviewee: No, the only thing I came across the link was in the package they gave me before surgery they mentioned something, a document sort of mentioning the prices and all that and they mentioned it in particular in regards to valve replacement. Having your dentist to check you out and making sure there is nothing wrong before they let you. That is the only time that I actually heard of any links between dental and cardiac

Facilitator: Aha, and what about after when you were in hospital or rehabilitation?

Interviewee: No, just actually here with you and this program. Apart from that document is the only time it has been mentioned

Facilitator: Ok, so you haven't heard. Is maintaining oral health important to you, is it a priority?

Interviewee: uhm…it probably hasn't being but about three or four…I was leaving overseas for a long time and I got into quite bad infected gums that was probably four of five years ago and so in the upper left corner I never floss before and sort of since then I floss pretty much at least every day or every second day. I try to floss because of that sort of problem I had with gums at that time

Facilitator: Did you need any treatment at the time?

Interviewee: I did. I couldn't because I was overseas. Then when I came back to Australia I saw a periodontist so I got it back to clear up the problem and I haven't had any problems since then

Facilitator: So he needed to do some treatment?

Interviewee: Yes I had to see him every two to three weeks for a couple of months, that was about four or five years ago

Facilitator: And form them you started to look after…

Interviewee: Yes I put more attention on them than I did in the past, yeah

Facilitator: So you said you floss…

Interviewee: Yeah flossing and brushing and actually I went to see the dentist about two weeks ago I had some pain there and he actually had to do some work with local anaesthetic, one of the gum has being infected that's all cleared up now and he gave me mouthwash and that's fine now

Facilitator: So that was two weeks ago when you saw the dentist

Interviewee: Yeah

Facilitator: Did he mention anything about the heart at the time?

Interviewee: No, actually he had to look up into the computer for research whether he could actually give me an anaesthetic or something because of the bypass or something about waiting for now or something, he had to do research. He was aware I had the bypass just recently and sort of had to do research about the effects of the anaesthetic

Facilitator: Oh, about someone who just had a bypass? More about how to treat you after your surgery and perhaps on some medications

Interviewee: Yeah

Facilitator: You said that you haven't received any information about it. If there is any, would you like to receive some and, if you would like to receive some, in which form would you like to receive it?

Interviewee: Probably just documents that I can read. Yeah got a bit to read something, some information about it

Facilitator: Ok, and when do you thing is the best time to talk about it if you think about your journey you know, being in hospital, seeing a doctor, coming to have surgery, coming out and then rehab. When do you think is better for you to receive information

Interviewee: Probably at the start of the rehab program in here because at the edge of having these problems and going to hospital I don't think it will change your habits while you are in hospital especially coming out of it. It will probably take a bit of time to change habits of a lifetime. Probably I think at the start of rehab it will probably be the best time

Facilitator: Are you talking about when they do the assessment like when the rehabilitation nurse did you assessment, that sort of time?

Interviewee: Yeah, probably the best time

Facilitator: Good, that's good. So, in relation to nurses, do you think nurses, cardiac nurses, can have a role in giving you information about oral health: looking at your mouth as initial assessment and refer you or suggest going to the dentist. Would you follow that advice?

Interviewee: I don't. I do not if they have a role or if they have the training. I can't answer that. If they give me advice to go to and see the dentist I will probably take it. Whether they are qualified to give advice I can't answer that question

Facilitator: I see. I was going to ask you next if you feel they are prepared enough. This is part of what we are trying to do: train the nurses to be able to do that. Incorporate it into cardiac rehabilitation if you think that is the best time and that is what other patients are saying they feel this is the best time. So we are preparing a program for that time because we think is the best time for what you are saying to us.

Interviewee: Ok

Facilitator: You mentioned you went to the dentist recently. Do you have any oral concerns now?

Interviewee: No, no is fine it fixed itself up so. I've got another annual check-up in about four months

Facilitator: Do you go every 6 months?

Interviewee: Every year

Facilitator: Is there any challenges for you going to the dentist or do you have any issues going to the dentist?

Interviewee: As in…

Facilitator: As in pain, difficulties getting there, cost?

Interviewee: I'm covered. I am in the defence force so all my medical treatment is covered by them so I can get in very easily most times to see a dentist

Facilitator: So you don't have to pay, that is good, lucky.

Interviewee: Yes

Facilitator: You mentioned that you work. Do you work full time, part time?

Interviewee: Full time

Facilitator:	So, basically access in not a problem, you drive, you are very independent in that sense
Interviewee:	I drive, yes
Facilitator:	You mentioned that you are ok receiving oral health information during cardiac rehabilitation
Interviewee:	Yes
Facilitator:	And you are comfortable with the nurses doing it and assessing you
Interviewee:	Yes, I feel comfortable
Facilitator:	Do you need you need any extra support looking after your oral health?
Interviewee:	No, no I'm very happy with the support I have
Facilitator:	You said you would follow the nurses' advice as well?
Interviewee:	yes, yes
Facilitator:	Do you think nurses have enough knowledge to provide information about oral health? You said you could not answer that
Interviewee:	Yes I suppose the question is whether cardiac nurses know anything about teeth and dental. Is a specialised field so…
Facilitator:	That right and that is the plan, we are planning to train the nurses. About social support, do you have social support?
Interviewee:	Yes family and friend
Facilitator:	And, are there any comments or suggestions in relation to what we are doing and the program we are planning to do?
Interviewee:	I suppose the questions is what I said about the relation between the dental the oral care and the heart. There is obviously a linkage, yes what impact it has on you know, I guess that will be part of the education
Facilitator:	Yes. When we finish the interview I will explain all that. If you have no other suggestions we are done with the interview. Thank you very much
